# Establishment of duplex multi-enzyme isothermal rapid amplification detection method for bovine astrovirus and norovirus

**DOI:** 10.3389/fvets.2026.1819282

**Published:** 2026-06-09

**Authors:** Lingling Jiang, Yuanfeng Zhao, Qin Zhang, Wenju Luo, Jingrui Zhou, Bo Yu

**Affiliations:** 1 Institute of Animal Husbandry and Veterinary Medicine, Guizhou Academy of Agricultural Sciences, Guiyang, China; 2 Guizhou Provincial Key Laboratory of Livestock and Poultry Genetic Resources Innovation and Utilization, Guiyang, China

**Keywords:** bovine astrovirus, bovine norovirus, diarrhea, duplex, multi-enzyme isothermal rapid amplification, rapid detection

## Abstract

In recent years, the detection rates of bovine astrovirus (BoAstV) and bovine norovirus (BNoV) in Chinese farms have continued to rise, resulting in significant economic losses to the livestock industry. To meet the need for rapid on-site screening, this study established a dual nucleic acid detection system capable of simultaneously detecting BoAstV and BNoV using the multi-enzyme isothermal rapid amplification technology. Its specificity, sensitivity, and repeatability were further validated using clinical samples. The experimental results showed that the established MIRA method specifically amplified the target nucleic acids of BoAstV and BNoV, while exhibiting no cross-reaction with other common diarrhea-associated pathogens, such as bovine rotavirus (BRV), bovine viral diarrhea virus, and bovine coronavirus, indicating good specificity. The method demonstrated high sensitivity, with detection limits of 920 copies/μL for BoAstV and 6,100 copies/μL for BNoV. In the repeatability tests, all the coefficients of variation were below 10%, confirming the high stability of the method. In clinical sample testing, the method showed strong agreement with conventional dye-based quantitative PCR results (Kappa value = 0.887), while offering shorter detection time and higher detection rates. In summary, the detection method established in this study demonstrated high sensitivity, strong specificity, and a short turnaround time, making it suitable for the rapid detection of bovine astrovirus and bovine norovirus in clinical samples of cattle.

## 1 Introduction

In accordance with the strategic directives of Guizhou Province to advance the high-quality development of the beef cattle industry, regional efforts to scale and modernize production are intensifying to scale and modernize production. Concurrently, the challenges associated with epidemic prevention and control have become increasingly pronounced. Viral diarrhea is a significant infectious disease threat in cattle, manifesting as diarrhea, reduced milk yield, immunosuppression, and potentially impairing the long-term growth performance of calves. Reports indicate that, alongside established pathogens such as bovine coronavirus (BCoV) and bovine viral diarrhea virus (BVDV), emerging agents such as bovine astrovirus (BoAstV) and bovine norovirus (BNoV) play an increasingly important role in the etiology of calf diarrhea (1–3). Consequently, establishing a rapid, sensitive, and field-deployable detection method is crucial for the early diagnosis and differential identification of diarrheal diseases.

Bovine astrovirus, a member of the *Astroviridae* family within the genus *Mammalian astrovirus*, is a single-stranded positive-sense RNA virus. First identified in diarrheic calf feces in the United Kingdom in 1978, it has since been reported in both healthy and diarrheic cattle in numerous countries (4). Global surveillance in 2015 revealed exceptionally high BoAstV detection rates in cases of viral diarrhea. Reported infection rates reached 87.5% in China, 85.7% in Japan, 66.7% in South Korea, 64.1% in Brazil, and 32.0% in Egypt (5). BoAstV infection is associated with clinical presentations, including diarrhea and neurological signs (6). Furthermore, BoAstV was detected in nasal swabs from cattle exhibiting respiratory symptoms, with a positivity rate of 8% (4, 7). In 2021, Zhu *et al*. reported a fecal detection rate of 46.34% in calves from 12 provinces in China, including Northeast, North, Central, Southwest, and Northwest China. Among them, the prevalence rate was 55.17% in diarrheic calves and 36.36% in asymptomatic calves (8). In 2019, Guangxi research reported that the infection rate of astrovirus in a buffalo farm was 40%, and the detection rate of feces was 11%. It was found that there was a recombination event with bovine astrovirus (BoAstV), and some strains were closely related to neurotoxicity-related strains, suggesting potential health risks (9). In 2023, Zhu et al. found through statistical analysis that the detection rate of BoAstv in diarrhea samples was 10.43%, which was also significantly higher than 7.69% in healthy cow dung samples, and showed synergistic pathogenicity with other bovine enteroviruses in diarrhea cow dung samples (10). Although astrovirus is not the main pathogen in calf diarrhea cases, it is one of the pathogens often detected in diarrhea samples because it often exists in mixed infections (11).

Bovine norovirus, a non-enveloped, single-stranded positive-sense RNA virus, belongs to the *Caliciviridae* family, genus *Norovirus*. All bovine noroviruses (BNoVs) belong to genogroup III (GIII), GIII consists of two genotypes: GIII.1 and GIII.2. The prototype strain was firstly isolated from diarrheic calves in the UK in 1976. Subsequently, BNoV has been widely detected in diarrheic calf feces across the Americas, Europe, Asia, and Africa (12–14). The first documented detection of BNoV in China occurred in 2017 (15), after which the virus has been widely disseminated within the country. Its pathogenic role in calf diarrhea is often underappreciated due to its frequent occurrence in co-infections with other diarrheal viruses (16). From 2017 to 2018, the positive rate of BNoV in diarrhea samples from six provinces of Sichuan, Xinjiang, Shaanxi, Liaoning, Shandong, and Henan in China was as high as 20.4% (17). BNoV was the only intestinal pathogen detected in the outbreak in Henan in 2018, suggesting its leading role in the outbreak (18). In 2025, the infection rate of bovine norovirus in Guangdong was 15.98%, which may be a new genetic variant of the Sichuan yak strain (19). In summary, BNoV has become an important cause of diarrhea in cattle in China.

Real-time fluorescence quantitative PCR (qPCR) is recognized internationally as the gold standard for virus detection, valued for its rapidity, simplicity, safety, high sensitivity, and quantitative capability, leading to its widespread adoption in clinical diagnostics (20). Although qPCR assays for the simultaneous detection of BoAstV and BNoV have been described (21–23), the typical assay duration remains approximately 1.5 h, comparable to that of conventional PCR, which limits its application in scenarios requiring rapid results. Advances in molecular biotechnology have led to the development of various convenient and efficient isothermal nucleic acid amplification technologies. Among these, Multi-enzyme Isothermal Rapid Amplification (MIRA), derived from Recombinase Polymerase Amplification (RPA), is a novel and efficient isothermal method. This technique relies on the synergistic action of four important proteins: RecA, gp41, single-stranded DNA-binding protein (SSB), and DNA polymerase, allowing for rapid DNA amplification at a constant temperature between 25 and 42 °C. The entire detection process can be completed within 30 min without the need for complex thermal cycling equipment, thereby simplifying the procedure (24–26).

Based on the high prevalence rate and mixed infection risk reported in many provinces in China, combined with the rapid development of the beef cattle industry in Guizhou but the lack of local monitoring data, the establishment of a rapid, sensitive, and stable BoAstV and BNoV dual method has a clear scientific basis and practical necessity. It not only improves the detection efficiency, but also reduces the cost. More importantly, it provides the epidemiological background data of the two viruses for the first time in Guizhou and even Southwest China, and provides technical support for the development of accurate prevention and control strategies. However, there is no rapid diagnosis method for BoAstV and BNoV in China. To address this gap, the present study designed specific primers and probes targeting the conserved genomic regions of BoAstV and BNoV. We successfully established a rapid, constant-temperature fluorescence detection method. This development provides a reliable technological tool and robust support for pathogen identification and molecular epidemiological investigations of bovine diarrhea.

## 2 Materials and methods

### 2.1 Viral nucleic acids and clinical samples

A total of 236 bovine fecal samples were collected from farms located in Guanling and Luodian counties, Guizhou Province. Positive viruses nucleic acid for Bovine Rotavirus (BRV), Bovine Viral Diarrhea Virus (BVDV), and Bovine Coronavirus (BCoV) were provided by the Physical and Chemical Detection and Analysis Laboratory for Livestock and Poultry Products at the Guizhou Provincial Institute of Animal Husbandry and Veterinary Medicine.

### 2.2 Nucleic acid extraction

Viral nucleic acids were extracted following the manufacturer’s instructions provided with the extraction kit. Total viral nucleic acid was isolated from the fecal swab samples, and the extracted products were stored at −80 °C for subsequent analysis. Fecal swab was mixed with 800 μL sterile saline, vortexed, and centrifuged at 3000 rpm for 5 min; 200 μL of the supernatant was then incubated with 10 μL of 10% SDS and 5 μL of 20 mg/mL proteinase K at 70 °Cfor 5 min, followed by nucleic acid release agent (RNA-II type) (Anpu Future Changzhou Biotechnology Co., Ltd., Changzhou, China) according to the manufacturer’s instructions. MIRA amplification using RNA thermostatic rapid amplification kit (fluorescent type) (Anpu Future Changzhou Biotechnology Co., Ltd., Changzhou, China).

In order to understand the potential PCR inhibitors in the fecal matrix, we used the experimental nucleic acid extraction method to detect the fecal spiked recovery test. We conducted spike-and-recovery experiments using fecal samples from healthy animals that tested negative for BoAstV and BNoV. Known concentrations of BoAstV and BNoV nucleic acids were spiked into the fecal matrix, extracted according to the previously outlined protocol, and quantified through TBGreen dye real-time PCR. A non-spiked fecal sample was used as a negative control. Recovery rates were calculated for each spiked concentration.

### 2.3 Probe and primers

Based on the BoAstV and BNoV gene sequences published in the GenBank database, multiple primer sets were designed to target the conserved regions of the BoAstV *ORF2* and BNoV *RdRp* genes using Oligo 6.0 software. The designed primers and probe sequences were subsequently synthesized by Guangzhou Aikerui Bioengineering Co., Ltd. (Guangzhou, China). BoAstV dye method amplified fragment 148 bp, BNoV dye method amplified fragment 80 bp. See Table 1 and Table 2 for details.

**Table 1:** Information on primer pairs and probes of BoAstV (HEX channel).

Method	Primer probe	Numbering	Sequence 5′-3’
MIRA	Upstream primer	AstV-F1	GAGGATGCAGATGATGAAGATGACGAGGTT
		AstV-F2	TGATGAATATTATGACATGCCACCTCTTGA
	Downstream primer	AstV-R1	AACTAACGCATCGTGGTACAGCCTGGTGAA
		AstV-R2	TTCGCTGTACTCATCGCTTGGTTTCAGCCG
		AstV-R3	GGGTGCAGCTCACAAAGGATCTCATAGGTT
	The original probe sequence	AstV-P	ACAGATGCCGACCTTGAGCTCGGTCCCATGGATGATTATGATGATCCACC
	Probe modification sequence		ACAGATGCCGACCTTGAGCTCGGTCCCATGGA/iHEXdT/idSp/A/iBHQ1dT/TATGATGATCCACC-C3 Spacer
TB Green	Upstream primer	148AstV-F	GGAGCCGCAGAGGTCATC
	Downstream primer	148AstV-R	CAACCGCAGCACCATTAACAG

**Table 2:** Information on primer pairs and probes of BNoV (FAM channel).

Method	primer	Sequence 5′-3′	Source
MIRA	Upstream primer	BNoV-F11	AACCGTAAGGTTCAGCTGCTCTGCCTCCTT
		BNoV-F22	CACAGCAACCGTAAGGTTCAGCTGCTCTGC
		BNoV-F3	TCCATGGTGACCGCAGAGGCCAAGGAA
	Downstream primer	BNoV-R1	TCCTGATTATCCTTGTCAGTCATCTTCATT
		BNoV-R2	AAATCGGGAAGGACGTCGCGACTACCTTCC
	The original probe sequence	BNoV-P	TCGCACCGCTCCATGTTTGCTTGGATGAGATTTCATGATTTGTCGCTGTG
	Probe modification sequence		TCGCACCGCTCCATGTTTGCTTGGATGAGATT/i6FAMdT/idSp/A/iBHQ1dT/GATTTGTCGCTGTG-C3 Spacer
TB Green	Upstream primer	80 BNoV-F	GACCAGGGTCCTCAAAGGGT
	Downstream primer	80 BNoV-R	GGTTGGGTCCGCGGGTCCAG

### 2.4 MIRA principle and condition setting

The MIRA fluorescence-based detection method used in this study employs a specific fluorescent probe with the modification pattern [FAM-dT][THF][BHQ1-dT]. During the reaction, the probe binds to the template strand, and the THF site is recognized and cleaved by the exonuclease present in the reagent. This cleavage separates the FAM fluorophore from the BHQ1 quencher, thereby releasing a detectable fluorescent signal. Amplification was performed by Real-Time PCR Instrument (Thermofisher ABI Q5, Thermo Fisher Scientific (China) Co., Ltd.). Detection channel and wavelength: FAM channel, with excitation/detection wavelength range of 490 nm–520 nm. Threshold determination method: Based on the background fluorescence level of negative controls and the default algorithm of the instrument, the threshold is set within the range of 10,000–20,000 (relative fluorescence units). Fluorescence recording mode: Recorded as real-time fluorescence curves, meaning fluorescence signals are continuously collected during the amplification process. Software for signal analysis: The accompanying QuantStudio Design & Analysis Software is used for fluorescence signal reading and data analysis.

The positive threshold is determined based on the negative control results within the same experimental run. Positive calls are considered valid only when the negative control results are normal. Specifically, the threshold line is set slightly above the fluctuation range of the negative amplification curves based on their morphology. This approach ensures that no Ct values are assigned to negative samples while enabling the reliable detection of amplification signals in positive samples. Each experimental run includes two negative control samples.

### 2.5 Preparation of recombinant plasmid standards

The two positive plasmids ASTV and BNOV were constructed separately according to the following methods. Construction of the BAstV recombinant plasmid: A diarrheic bovine fecal sample collected from a cattle farm in Nayong County, Guizhou Province in 2024 was used as the starting material. The sample was confirmed as BAstV-positive by RT-PCR using the primers designed in this study, followed by sequencing. The nucleic acid from this positive sample was extracted using the method established in this study and served as the template. Specific primers obtained from screening were used for amplification. The PCR product with the expected band size and good specificity was identified by 2% agarose gel electrophoresis, and the target fragment was recovered using a gel extraction kit (Tiangen Biotech Co., Ltd., Beijing, China). The purified target fragment was ligated into the pMD18-T cloning vector and transformed into competent DH-5α cells. The resulting recombinant plasmid was designated pMD-18 T-BAstV and sent to Shanghai Sangon Biotech Co., Ltd. for sequencing verification. After confirmation of positivity by sequencing, the recombinant bacteria were cultured at scale, and the pMD-18 T-BAstV plasmid was extracted using a plasmid extraction kit. Its concentration was determined using NanoDrop Eight Microvolume UV–Vis Spectrophotometer (Thermo Fisher Scientific Technology Co., Ltd., China). Construction of the BNoV recombinant plasmid: The same method as for BAstV was used to construct the pMD-18 T-BNoV recombinant plasmid, using a local suspected sample confirmed as BNoV-positive by sequencing as the template.

### 2.6 Amplified reaction reaction system

MIRA reaction: Two plasmid concentrations of 100 fg/μL were used as templates, respectively., a 50 μL reaction system was assembled according to the instructions of the RNA isothermal rapid amplification fluorescence kit: 29.4 μL of Buffer A, 2.0 μL each of upstream and downstream primers (10 μM), 0.6 μL of probe, 5 μL of template, 2.5 μL of Buffer B, and ddH₂O were added to a final volume of 50 μL. A reaction containing ddH_2_O instead of the template was used as a negative control. Amplification was performed under the following conditions: incubation at 39 °C for 30 s, repeated for 40 cycles.

TBgreen real-time PCR reaction system: TB Green® *Premix Ex Taq*™ II Mix 12.5 μL (TaKaRa Biotechnology Co., Ltd. (Dalian, China)), upstream primers 1 μL, downstream primers 1 μL, positive plasmids 2 μL, and ddH₂O were added to a final volume of 25 μL. Reaction condition: 95 °C 30s, 95 °C 5 s, 60 °C 35 s. The last two steps of 40 cycles. Real-PCR amplification is carried out on the Thermofisher QuantStudio™ 1 Plus (Thermo Fisher Scientific (China) Co., Ltd).

### 2.7 Optimization of primer screening for singleplex multi-enzyme isothermal fluorescence amplification

The two recombinant plasmids were serially diluted to concentrations of 100 fg/μL, 10 fg/μL, and 1 fg/μL, respectively. The specific dilution concentration of the plasmid is shown in the results. The forward and reverse primers listed in Table 3 was systematically paired for both positive and negative screening assays. Simultaneously, negative control reaction was performed using ddH_2_O instead of the template. The ddH_2_O used in the negative control in this research was set up with two replicates. Isothermal fluorescence amplification was conducted under standardized conditions. The optimal primer combination was selected based on the amplification curve characteristics, specifically evaluating the time to peak fluorescence signal and the relative fluorescence intensity.

**Table 3:** Screening results of BoAstV primers.

Screening methods	Upstream primers	Downstream primers	Probe	CT value			
				100 fg/μL	10 fg/μL	1 fg/μL	N
				9.2 × 10^3^copies/μL	9.2 × 10^2^copies/μL	9.2 × 10^1^copies/μL	N
Positive screening	AstV-F1	AstV-R1	AstV-P	13.65/14.85	NoCt/NoCt	NoCt/NoCt	NoCt/NoCt
		AstV-R2		13.55/13.34	NoCt/NoCt	32.34/36.82	NoCt/NoCt
		AstV-R3		8.09/7.35	NoCt/19.73	31.13/NoCt	NoCt/NoCt
Reverse screening	AstV-F1	AstV-R2		13.55/13.34	NoCt/NoCt	32.34/36.82	NoCt/NoCt
		AstV-R3		8.09/7.35	NoCt/19.73	31.13/NoCt	NoCt/NoCt
	AstV-F2	AstV-R2		17.26/17.60	NoCt/36.83	NoCt/NoCt	NoCt/NoCt
		AstV-R3		12.22/12.40	NoCt/34.27	24.66/24.60	NoCt/NoCt

### 2.8 Sensitivity evaluation of the uniplex multi-enzyme isothermal fluorescence reaction

The recombinant plasmids of the two viruses were double-diluted to create four gradient concentrations: 100 fg/μL, 10 fg/μL, 1 fg/μL, and 0.1 fg/μL, which served as templates for the amplification reaction. ddH₂O was used as a negative control. The sensitivity of the singleplex reaction was assessed, and the optimal primer combination was selected based on the results.

### 2.9 Optimization of primer concentration for duplex multi-enzyme isothermal fluorescence amplification

The intrinsic fluorescence intensity of the HEX fluorophore is relatively low because of its inherent properties, whereas the FAM fluorophore exhibits a brighter signal emission. To balance the fluorescence intensity and sensitivity of the dual detection system, the concentration of the FAM-labeled primer-probe for BNoV was optimized. Accordingly, the duplex fluorescence reaction was adjusted by varying the amount of the BNoV primers. Using the recombinant plasmid as the template, a 50 μL reaction was assembled according to the instructions of the RNA isothermal rapid amplification fluorescence kit, containing: 29.4 μL of Buffer A; 1.5 μL each of BoAstV primers BoAstV-F1 and BoAstV-R3 (10 μM); 0.6 μL of BoAstV probe BoAstV-P (10 μM); 1.0 μL or 1.2 μL of BNoV primer BNoV-F3 (10 μM) and 1.2 μL or 1.0 μL of BNoV-R1 (10 μM), respectively; 0.3 μL or 0.4 μL of BNoV probe BNoV-P (10 μM); 5 μL of template; 2.5 μL of Buffer B; and ddH₂O added to a final volume of 50 μL. A reaction with ddH₂O instead of the template served as the negative control. Amplification was conducted at 39 °C for 30 s per cycle, for a total of 40 cycles.

### 2.10 Sensitivity evaluation of the duplex multi-enzyme isothermal fluorescence reaction

After determining the nucleic acid concentration, the recombinant plasmid was serially diluted into four gradients and used as a template for PCR amplification. The negative control reaction was performed using ddH_2_O. Each dilution was tested in duplicate to evaluate the sensitivity of the proposed method.

### 2.11 Specificity evaluation of the duplex multi-enzyme isothermal fluorescence reaction

The specificity of the established method was assessed by testing it against common bovine diarrhea viruses nucleic acid, including Bovine Rotavirus (BRV), Bovine Viral Diarrhea Virus (BVDV), and Bovine Coronavirus (BCoV). Concurrently, the recombinant plasmids pMD-18 T-BoAstV and pMD-18 T-BNoV were diluted to a concentration of 10 pg./μL and used as positive controls, while ddH₂O served as the negative control to verify the assay specificity.

### 2.12 Repeatability evaluation of the duplex multi-enzyme isothermal fluorescence reaction

The repeatability of the assay was evaluated by performing multiple amplification reactions using the recombinant plasmid at serial dilution levels of 1 pg./μL, 100 fg/μL, 10 fg/μL. The repeatability test was carried out by using two reagents to test the same template synchronously (two independent reaction systems were set up for each sample in each experiment). The intra-batch and inter-batch errors of the reagent of the manufacturer were controlled within 2.5%. The verification experiment was completed independently by an operator. A total of three batches were tested (48 h interval per batch), and each batch was repeated twice. The specific dilution copy number of the two plasmids is shown in the results.

### 2.13 Clinical sample detection

A total of 236 bovine fecal samples were collected and tested using the duplex isothermal RT-PCR method established in this study. The results were compared with those obtained from the TB Green dye-based PCR method to analyze the prevalence and co-infection patterns of BoAstV and BNoV.

## 3 Results

### 3.1 Recombinant plasmid concentration

The constructed recombinant plasmids were quantified according to the methodology described in this article. The concentrations of recombinant plasmids pMD-18 T-BoAstV and pMD-18 T-BNoV were 197 ng/μL and pMD-18 T-BNoV was 241.7 ng/μL, respectively.

### 3.2 Primer screening

#### 3.2.1 Screening results of BoAstV primers

##### 3.2.1.1 BoAstV primer screening

**Figure 1:**
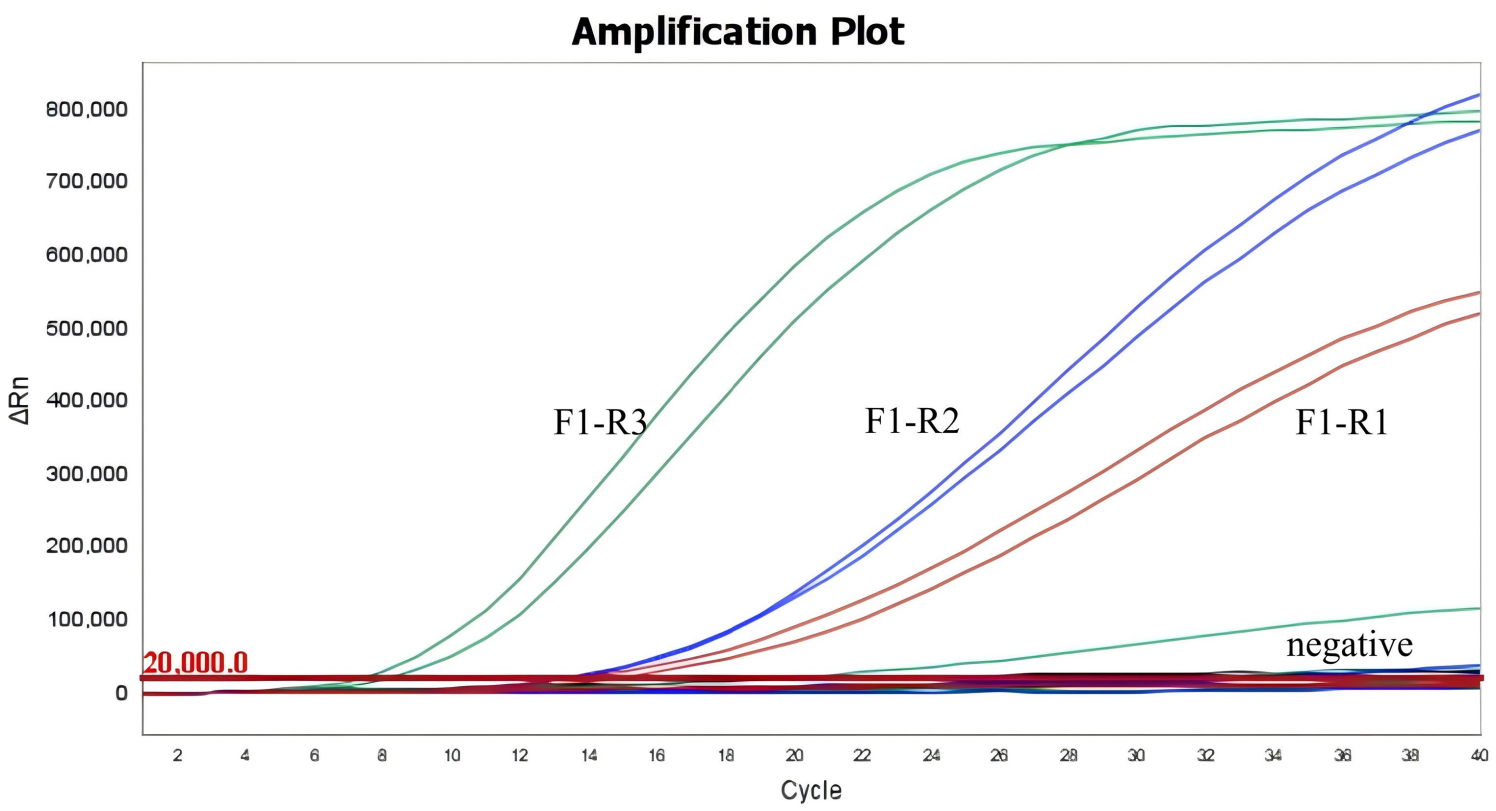
BoAstV primer screening results. The upstream primers were F1, and the downstream primers were R1–R3. Two replicates for each sample.

From Figure 1 and Table 3, it shows that when the BoAstV plasmid was 9.2 × 10^3^ copies/μL (100 fg/μL), the upstream primer was F1, and the downstream primers were R1, R2, and R3, respectively. The F1-R3 primer combination has the smallest CT, the F1-R2 primer combination is stable, and the F1-R1 primer combination is slow and unstable. Therefore, the downstream primers R3 and R2 are selected for the next selection.

##### 3.2.1.2 BoAstV primer reverse screening

**Figure 2:**
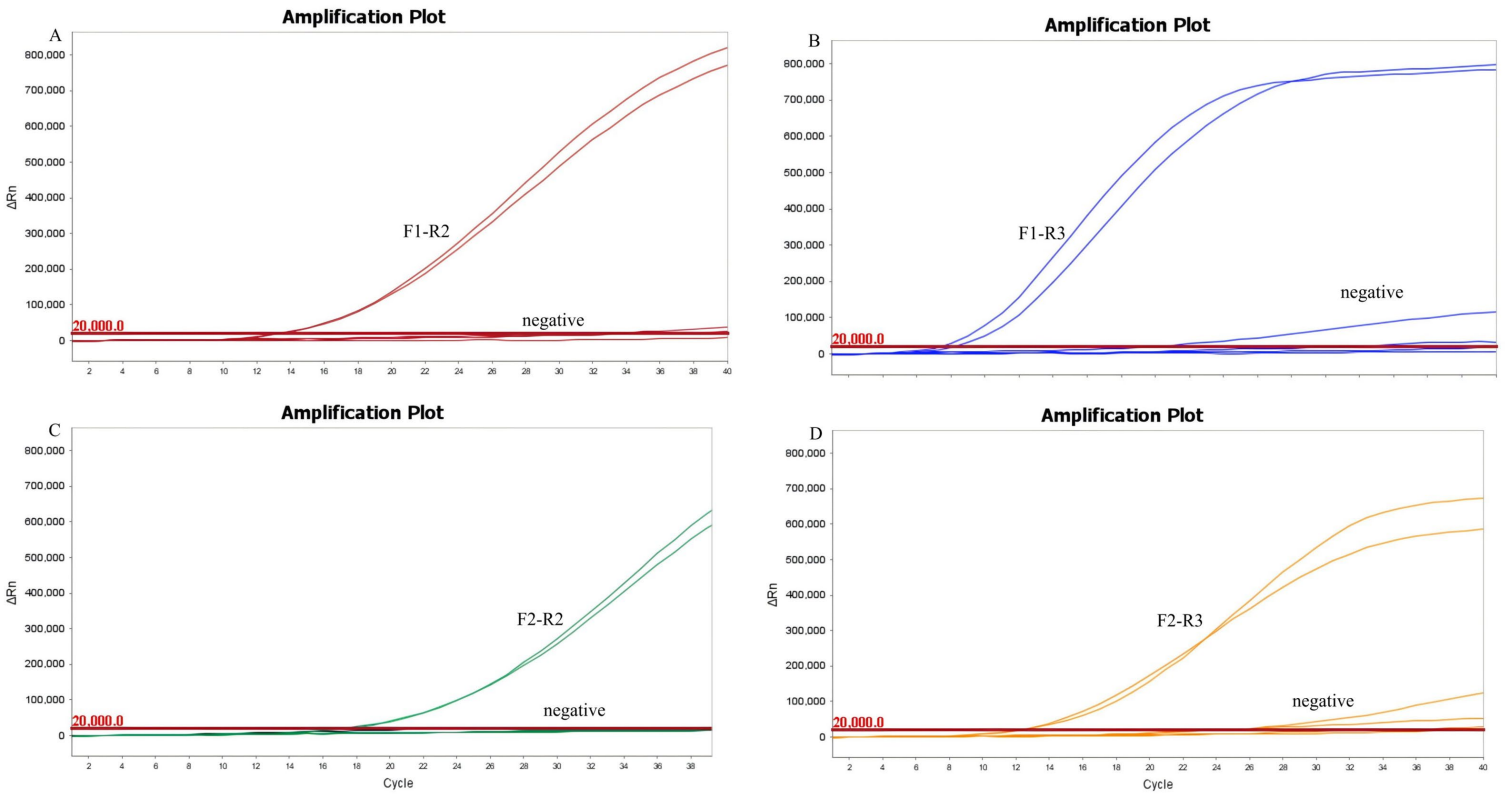
BoAstV primer reverse screening **(A–D)**. **(A)** Upstream primer F1 and downstream primer R2. **(B)** Upstream primer F1 and downstream primer R3. **(C)** Upstream primer F2 and downstream primer R2. **(D)** Upstream primer F2 and downstream primer R3.

Reverse screening results indicated that the primer-probe set AstV-F1 + AstV-R3 + AstV-P yielded the lowest CT value and the highest fluorescence intensity across all three template concentrations, thus representing the optimal combination (Table 3; Figure 2).

#### 3.2.2 Screening results of BNoV primers

##### 3.2.2.1 BNoV primer screening

**Figure 3:**
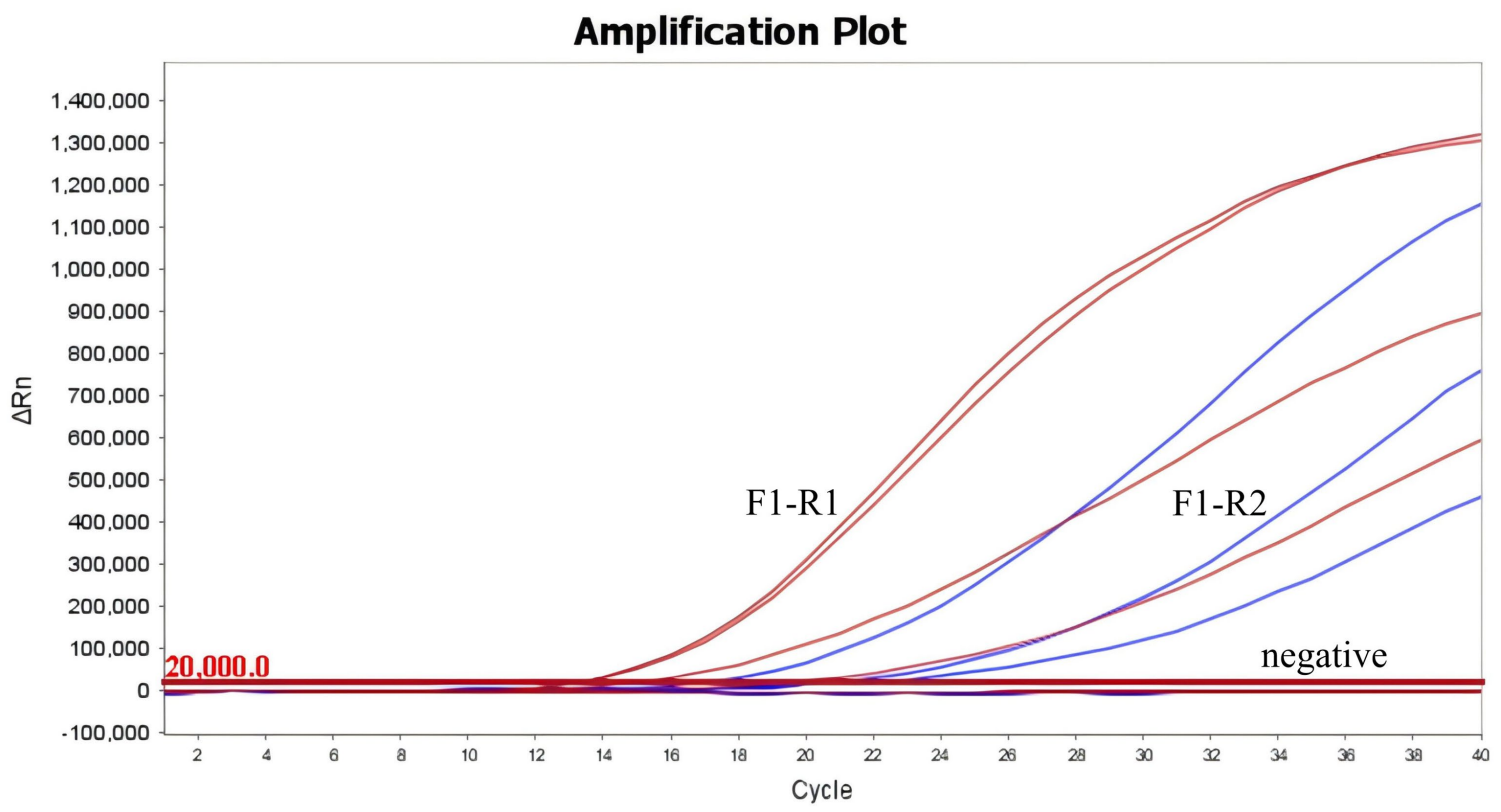
BNoV primer screening results. Red: upstream primer F1 and downstream primer R1; blue: upstream primer F1 and downstream primer R2. Two replicates for each sample.

As shown in Figure 3 and Table 4, when the downstream primer was R1, the CT value was the smallest when the plasmid concentration was 6.1 × 10^3^ copies/μL (100 fg/μL), and the detected CT value was relatively low when the plasmid concentration was 6.1 × 10^2^ copies/μL (10 fg/μL) and 61 copies/μL (1 fg/μL).

**Table 4:** Screening results of BNoV primers.

Screening methods	Upstream primers	Downstream primers	Probe	CT value			
				100 fg/μL	10 fg/μL	1 fg/μL	N
				6.1 × 10^3^copies/μL	6.1 × 10^2^copies/μL	6.1 × 10^1^copies/μL	N
Positive screening	BNoV-F1	BNoV-R1	BNoV-P	13.27/13.20	14.93/NoCt	NoCt/19.17	NoCt/NoCt
		BNoV-R2		20.75/16.84	21.11/NoCt	NoCt/NoCt	NoCt/NoCt
Reverse screening	BNoV-F1	BNoV-R1		13.27/13.20	14.93/NoCt	NoCt/19.17	NoCt/NoCt
	BNoV-F2			15.84/16.06	21.81/NoCt	NoCt/NoCt	NoCt/NoCt
	BNoV-F3			13.98/12.83	13.05/31.69	NoCt/NoCt	NoCt/NoCt

##### 3.2.2.2 Reverse screening of BNoV primers

**Figure 4:**
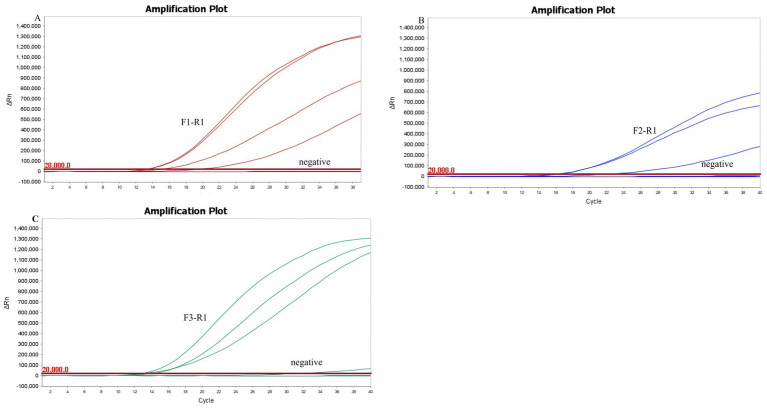
BNoV primer reverse screening **(A–C)**. **(A)** Upstream primer F1 and downstream primer R1. **(B)** Upstream primer F2 and downstream primer R1. **(C)** Upstream primer F3 and downstream primer R1. Two replicates for each sample.

As shown in Table 4 and Figure 4, at each of the three template concentrations, the primer-probe combination BNoV-F3 + BNoV-R1 + BNoV-P exhibited the lowest CT value and the highest fluorescence intensity, indicating that it was the optimal combination. The combination of BNoV-F1 + BNoV-R1 + BNoV-P was the second-best option. Therefore, these two combinations were selected for subsequent sensitivity testing to identify the optimal primer set.

### 3.3 Sensitivity detection of uniplex multi-enzyme isothermal fluorescence reaction

#### 3.3.1 Sensitivity detection of BoAstV multi-enzyme isothermal fluorescence reaction

**Figure 5:**
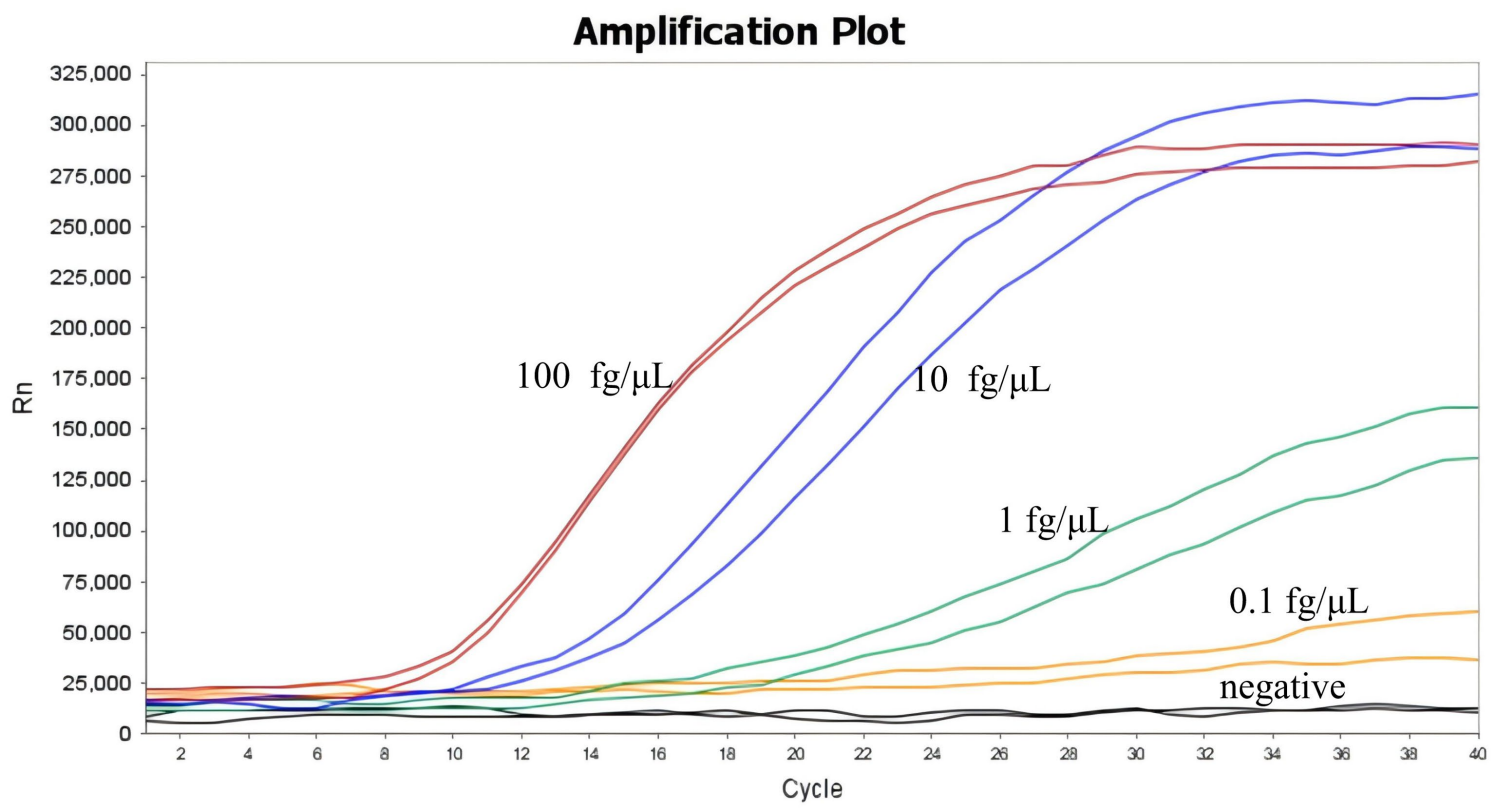
Sensitivity of BoAstV MIRA (upstream primer F1 and downstream primer R3). The BoAstV plasmid concentrations are 9.2 × 10^3^ copies/μL (100 fg/μL), 9.2 × 10^2^ copies/μL (10 fg/μL), 92 copies/μL (1 fg/μL), and 9.2 copies/μL (0.1 fg/μL), respectively. Two replicates for each sample.

As shown in Table 5 and Figure 5, the combination of BoAstV-F1 + BoAstV-R3 demonstrated a sensitivity of 92 copies/μL (1 fg/μL) and was identified as the optimal primer-probe set.

**Table 5:** Sensitivity results of uniplex MIRA of BoAstV and BNoV.

Virus	Upstream primers	Downstream primers	Probe	CT value				
				100 fg/μL	10 fg/μL	1 fg/μL	0.1 fg/μL	N
BoAstV	BoAstV-F1	BoAstV-R3	BoAstV-P	9.2 × 10^3^ copies/μL	9.2 × 10^2^ copies/μL	9.2 × 10^1^ copies/μL	9.2 copies/μL	N
				9.98/10.01	13.61/12.77	20.57/19.09	32.39/NoCt	NoCt/NoCt
BNoV	BNoV-F1	BNoV-R1	BNoV-P	6.1 × 10^3^ copies/μL	6.1 × 10^2^ copies/μL	6.1 × 10^1^ copies/μL	6.1 copies/μL	N
				11.68/11.08	15.13/14.76	23.64/20.29	35.81/NoCt	NoCt/NoCt
	BNoV-F3			10.11/11.10	12.67/15.26	20.35/17.67	35.82/36.25	NoCt/NoCt

#### 3.3.2 Sensitivity detection of BNoV multi-enzyme isothermal fluorescence reaction

**Figure 6:**
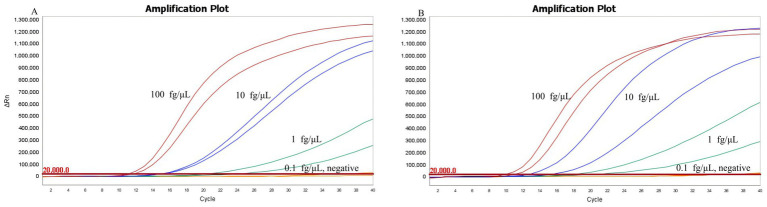
Sensitivity of BNoV MIRA **(A,B)**. **(A)** Upstream primer F1 and downstream primer R1. **(B)** Upstream primer F3 and downstream primer R1. The BNoV plasmid concentrations of 6.1 × 10^3^ copies/μL (100 fg/μL), 6.1 × 10^2^ copies/μL (10 fg/μL), 61 copies/μL (1 fg/μL), and 6.1 copies/μL (0.1 fg/μL) respectively. Two replicates for each sample.

The results in Table 5 and Figure 6 show that the sensitivity of the BNoV-F1 + BNoV-R1 and BNoV-F3 + BNoV-R1 primers was 61 copies/μL (1 fg/μL), and BNoV-F3 + BNoV-R1 was the optimal primer-probe combination.

### 3.4 Duplex multi-enzyme isothermal fluorescence reaction primer dosage optimization

#### 3.4.1 Optimization scheme 1 for duplex multi-enzyme isothermal fluorescence sensitivity (BNoV forward and reverse primers at 1 μL each)

**Figure 7:**
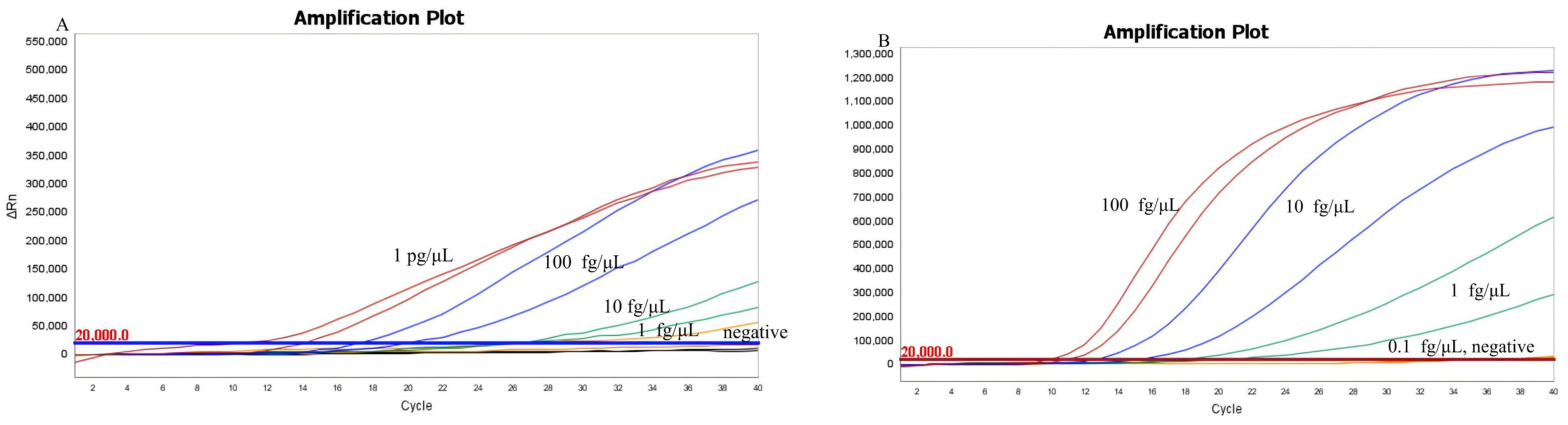
Duplex MIRA primer dosage scheme 1 reaction diagram **(A,B)**. The primers for BoAstV were utilized at a volume of 1.5 μL, while the primers for BNoV were used at 1 μL. **(A)** For BoAstV, the plasmid concentrations were as follows: 9.2 × 10^4^ copies/μL (1 pg./μL), 9.2 × 10^3^ copies/μL (100 fg/μL), 9.2 × 10^2^ copies/μL (10 fg/μL), and 9.2 × 10^1^ copies/μL (1 fg/μL). **(B)** For BNOV, the plasmid concentrations were as follows: 6.1 × 10^4^ copies/μL (1 pg./μL), 6.1 × 10^3^ copies/μL (100 fg/μL), 6.1 × 10^2^ copies/μL (10 fg/μL), 6.1 × 10^1^ copies/μL (1 fg/μL). Two replicates for each sample.

The results from the duplex optimization scheme 1 demonstrated that when 1 μL each of BNoV forward and reverse primers were used along with 0.3 μL of BNoV probe, the sensitivities achieved were 920 copies/μL (10 fg/μL) for BoAstV and 6,100 copies/μL (100 fg/μL) for BNoV (Table 6 and Figure 7).

**Table 6:** The results of two optimization schemes of BoAstV-BNoV duplex MIRA.

Scheme	Upstream primers	Downstream primers	Probe	CT value				
				1 pg./μL	100 fg/μL	10 fg/μL	1 fg/μL	N
Scheme 1	BoAstV			9.2 × 10^4^copies/μL	9.2 × 10^3^copies/μL	9.2 × 10^2^copies/μL	9.2 × 10^1^copies/μL	N
	F1 (1.5 μL)	R3 (1.5 μL)	AstV-P (0.6 μL)	10.24/14.16	17.36/20.22	25.84/26.90	NoCt/28.03	NoCt/NoCt
	BNoV			6.1 × 10^4^copies/μL	6.1 × 10^3^copies/μL	6.1 × 10^2^copies/μL	6.1 × 10^1^copies/μL	N
	BNoV-F3 (1μL)	BNoV-R1 (1μL)	BNoV-P (0.3 μL)	18.61/21.66	23.98/28.92	33.69/39.51	NoCt/NoCt	NoCt/NoCt
Scheme 2	BoAstV			9.2 × 10^4^copies/μL	9.2 × 10^3^copies/μL	9.2 × 10^2^copies/μL	9.2 × 10^1^copies/μL	N
	F1 (1.5 μL)	R3 (1.5 μL)	AstV-P (0.6 μL)	14.65/16.15	21.57/19.90	29.48/32.73	33.14/33.27	NoCt/NoCt
	BNOV			6.1 × 10^4^copies/μL	6.1 × 10^3^copies/μL	6.1 × 10^2^copies/μL	6.1 × 10^1^copies/μL	N
	BNoV-F3 (1.2 μL)	BNoV-R1 (1.2 μL)	BNoV-P (0.4 μL)	20.31/23.32	27.76/25.62	37.34/NoCt	NoCt	NoCt/NoCt

#### 3.4.2 Optimization scheme 2 for duplex multi-enzyme isothermal fluorescence sensitivity (BNoV forward and reverse primers at 1.2 μL each)

**Figure 8:**
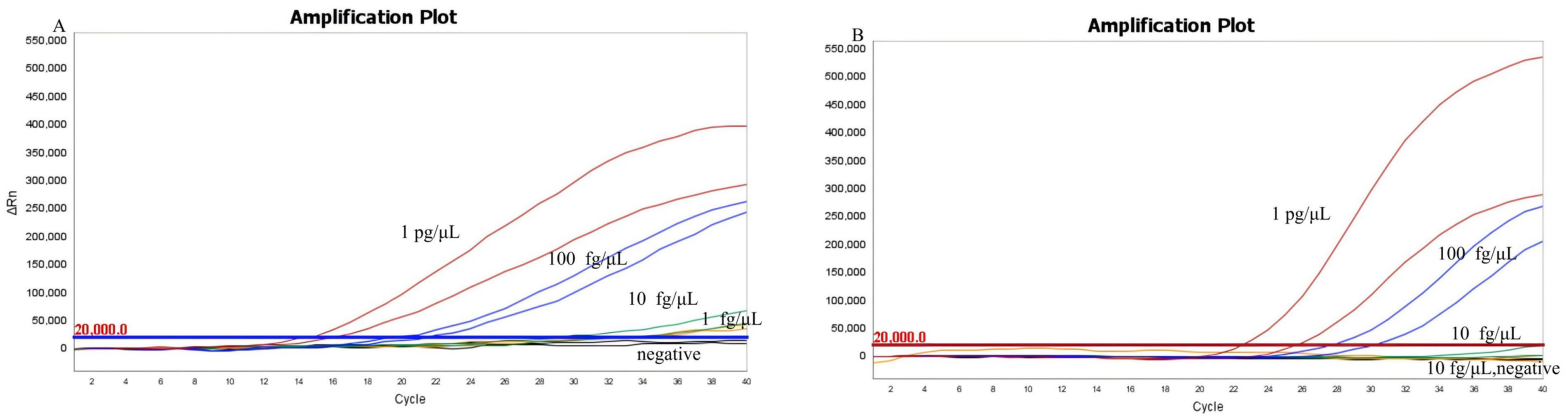
Duplex MIRA primer dosage scheme two reaction diagram **(A,B)**. The primers for BoAstV were used at 1.5 μL, BNoV primers for 1.2 μL. **(A)** For BoAstV. The concentration of plasmid 9.2 × 10^4^ copies/μL (1 pg./μL), 9.2 × 10^3^ copies/μL (100 fg/μL), 9.2 × 10^2^ copies/μL (10 fg/μL), 9.2 × 10^1^ copies/μL (1 fg/μL). **(B)** For BNOV. The concentration of plasmid 6.1 × 10^4^ copies/μL (1 pg./μL), 6.1 × 10^3^ copies/μL (100 fg/μL), 6.1 × 10^2^ copies/μL (10 fg/μL), 6.1 × 10^1^ copies/μL (1 fg/μL). All samples were two replicates.

As shown in Table 6 and Figure 8, the results from duplex optimization scheme 2 demonstrated a sensitivity of 920 copies/μL (10 fg/μL) for BoAstV and 6,100 copies/μL (100 fg/μL) for BNoV.

The results showed that after increasing the dosage of BNoV primers, the CT values of the two samples at the same concentration differed slightly, which was more stable than that of Scheme One. Therefore, option Two is chosen, that is, the amounts of primers upstream and downstream of BoAstV are 1.5 μL respectively, the amount of BoAstV-P is 0.6 μL, and the detection sensitivity reaches 920 copies/μL (10 fg/μL). The primers used upstream and downstream of BNoV were 1 μL each, the dosage of BNOV-P was 0.3 μL, and the detection sensitivity reached 6,100 copies/μL (100 fg/μL).

### 3.5 Specificity of the duplex multi-enzyme isothermal fluorescence assay

**Figure 9:**
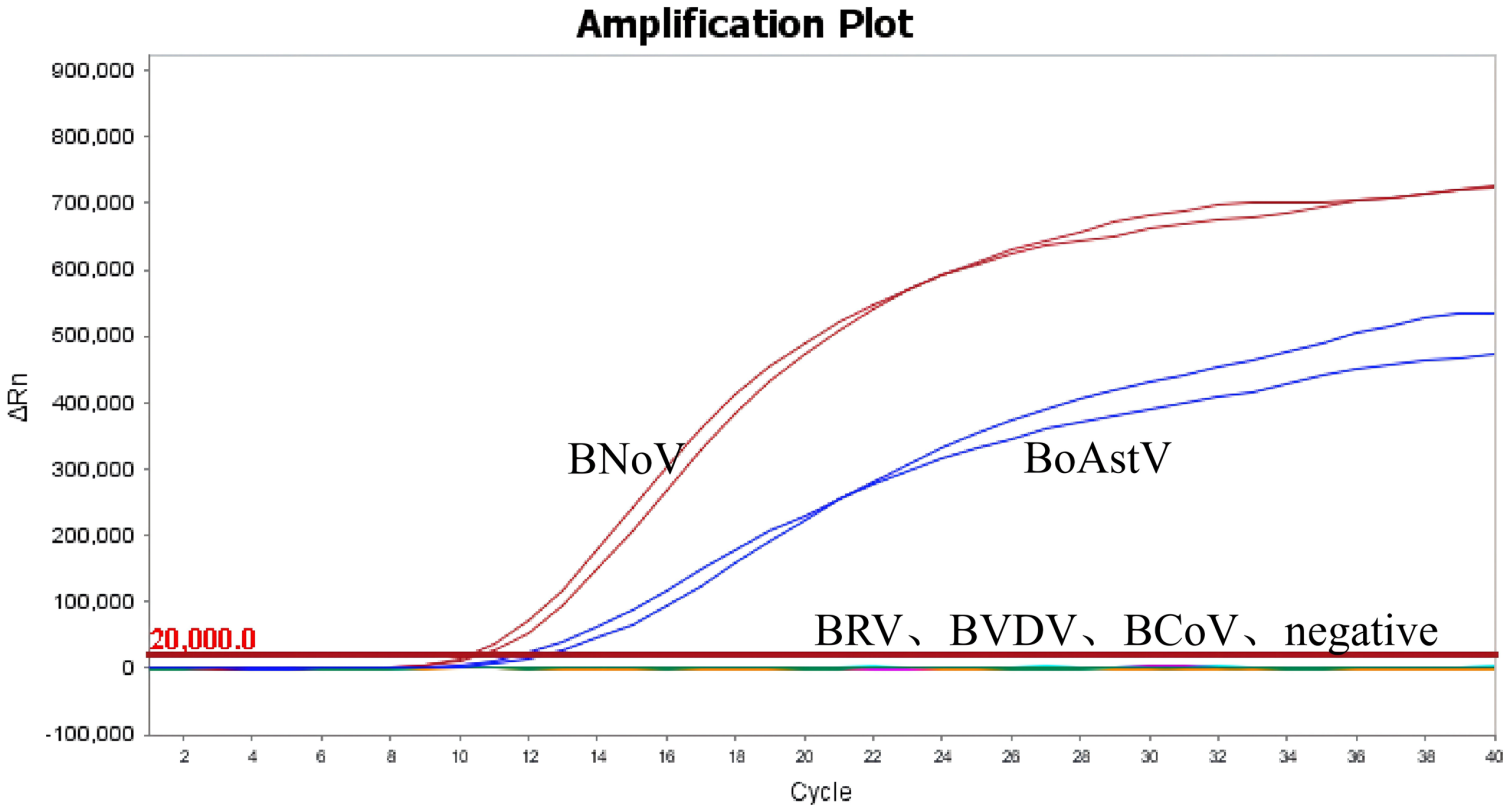
The specificity of duplex multi-enzyme isothermal fluorescence reaction. Red line represents BNoV plasmid and blue line represents BoAstV plasmid. Other lines are nucleic acid of Bovine Rotavirus (BRV), Bovine Viral Diarrhea Virus (BVDV), Bovine Coronavirus (BCoV), and ddH_2_O. Two replicates for each sample.

To evaluate the specificity of the assay, common diarrheal pathogens in cattle, including common viral plasmids Bovine Rotavirus (BRV), Bovine Viral Diarrhea Virus (BVDV), and Bovine Coronavirus (BCoV), were tested simultaneously. The results showed that only the recombinant plasmids pMD-18 T-BoAstV and pMD-18 T-BNoV produced positive signals, while no cross-reactivity was observed with the other pathogens (Figure 9). This confirmed that the established duplex assay exhibited high specificity.

### 3.6 Reproducibility of the fuplex multi-enzyme isothermal fluorescence assay

To assess the reliability of the assay, the established isothermal fluorescence method was used to repeatedly test recombinant plasmids at different concentrations in four independent experiments. As shown in Table 7, the coefficient of variation (CV) of the Ct values at each concentration was below 10%, indicating low dispersion. These results demonstrate that the developed duplex fluorescence detection method is reproducible.

**Table 7:** Repeatability test results.

Virus	Plasmid standard	Intra-assay variability		Inter-assay variability	
	copies/μL	Ct (x̄±S)	*CV*%	Ct (x̄±S)	*CV*%
BoAstV	9.2 × 10^4^	15.40 ± 1.06	6.89	14.22 ± 1.26	8.84
	9.2 × 10^3^	20.74 ± 1.18	5.70	19.52 ± 1.42	7.27
	9.2 × 10^2^	31.11 ± 2.30	7.39	28.47 ± 2.51	8.81
BNOV	6.1 × 10^4^	21.82 ± 2.13	9.76	20.66 ± 1.63	7.88
	6.1 × 10^3^	26.69 ± 1.51	5.67	26.61 ± 2.04	7.67
	6.1 × 10^2^	36.88 ± 1.22	3.32	34.43 ± 2.63	7.65

### 3.7 Clinical sample testing

#### 3.7.1 Spike-and-recovery experiments of fecal samples

When the standard nucleic acid addition concentration was 59.98 ng/μL, the recovery rate was 33.37%, and the addition amount was 41.84 ng/μL, the recovery rate was 12.14%. The results indicated a concentration-dependent reduction in recovery at lower spiked nucleic acid levels, suggesting the presence of a mild inhibitory effect. Furthermore, since our primary objective was to compare the developed rapid dual-detection method with the laboratory-standard real-time PCR, any matrix-induced bias (if existed) would likely impact both methods similarly. Therefore, this bias would not compromise the validity of our comparative conclusions.

#### 3.7.2 Detection of clinical samples by MIRA method

As shown in Table 8, fluorescent dye-based RT-qPCR detected 54 BoAstV-positive samples, 19 BNoV-positive samples, and four co-infected samples. In comparison, the duplex isothermal PCR method detected 55 BoAstV-positive, 21 BNoV-positive, and five co-infected samples. The results of the two methods were highly consistent, with Kappa values of 0.988 for BoAstV, 0.988 for BNoV, and 0.887 for the detection of both viruses. These findings indicate that the duplex multi-enzyme isothermal method offers high detection rates, strong specificity, and a rapid turnaround time of only 20 min. Therefore, the established method is suitable for rapid clinical sample detection. The results of TB Green dye-based PCR showed that the CV value of intra-batch repeated test was not more than 0.84%, the CV value of inter-batch repeated test was not more than 6.81%, and the coefficient of variation within and between groups was less than 10%. The established double detection method met the requirements (Supplementary Table 1; Supplementary Figures 1–6).

**Table 8:** Results of clinical sample testing.

Pathogens	Number	TB Green RT-PCR		MIRA fluorescence quantitative PCR		Kappa
		Positive	Positive rate%	Positive	Positive rate%	
BoAstV	236	54	22.88	55	23.31	0.988
BNoV	236	19	8.05	21	8.90	0.945
BoAstV+BNoV	236	4	1.69	5	2.12	0.887

## 4 Discussion

Calf diarrhea is a major digestive disorder in the cattle breeding industry, with infectious agents being a significant contributing factor (27). The etiology involves various pathogens, including viruses, bacteria, and mycoplasmas (28). Given the diversity of causative agents, accurate pathogen identification is key to effective prevention and control, highlighting the growing research focus on viral pathogens as key etiological agents.

Advances in molecular biotechnology have led to an increased detection rate of emerging viruses in cattle, such as BoAstV and BNoV, in China (18). Traditional diagnostic methods, such as virus isolation and electron microscopy, are often labor-intensive and lack specificity. In contrast, PCR-based techniques are widely adopted due to their convenience and efficiency. Although real-time quantitative PCR offers high sensitivity, it requires a controlled laboratory environment, is susceptible to aerosol contamination, and involves prolonged processing time. Among isothermal amplification technologies, loop-mediated isothermal amplification (LAMP) carries the risk of aerosol contamination, requires a PCR laboratory setup, and has a relatively high false-positive rate (26). Recombinase polymerase amplification (RPA) is associated with high costs and extended supply cycles. Domestically developed recombinase-aided amplification (RAA) still faces challenges in terms of interference resistance and manufacturing consistency (29). In comparison, multi-enzyme isothermal rapid amplification (MIRA) employs a multi-enzyme system that demonstrates strong anti-interference capability and high stability (30). MIRA technology has been successfully applied to detect various animal diseases (31, 32).

A duplex PCR method for detecting bovine astrovirus (BoAstV) and bovine norovirus (BNoV) has been previously established and applied. Both BoAstV and BNoV are RNA viruses. In this study, after pre-treatment with SDS and proteinase K, the nucleic acid release agent (RNA-II) kit was used to quickly extract nucleic acid in 15 min (3).

Compared with the traditional polymerase chain reaction (PCR), MIRA technology has the advantages of short reaction time, simple operation, and no need for precise temperature control equipment. It is especially suitable for on-site rapid detection in resource-limited areas technology can complete nucleic acid amplification in 15–30 min through the synergistic effect of multiple enzymes under constant temperature conditions, while ordinary PCR usually takes 1–2 h (3).

Studies have shown that the detection sensitivity of MIRA is usually higher than that of ordinary PCR, up to 10–1,000 times. For example, in the detection of *S. sinensis,* the detection limit of MIRA was 10 pg./L, while that of PCR was only 1 ng/L (33). However, in some cases, the sensitivity of MIRA may be slightly lower than that of real-time fluorescent quantitative PCR (qPCR). For example, when detecting *Aeromonas hydrophila,* the detection limit of MIRA is 1 fg/μL, while qPCR is 0.1 fg/μL (32). In general, the sensitivity of MIRA was comparable to or slightly lower than that of qPCR, but significantly better than that of conventional PCR. Many studies have confirmed that the MIRA method can specifically detect target pathogens. For example, MIRA can specifically detect Porcine circovirus type 4 (PCV4), PCV2, PCV3, and PPV without cross-reaction. MIRA-LFD can specifically detect duck plague virus, and has no cross-reaction with avian adenovirus and goose astrovirus (3).

The most prominent advantage of MIRA is its reaction speed. Common PCR usually takes 1–2 h to complete amplification, while MIRA can be completed within 15–30 min (34). More importantly, MIRA is performed under constant temperature conditions (usually 35–45 °C) without the need for variable temperature cycling equipment required for PCR (35). For example, MIRA can work efficiently at constant temperatures such as 37 °C, 39 °C or 42 °C (36). In addition, by optimizing the reaction conditions, the detection threshold was successfully set, and the accuracy of detection was improved (37). This makes MIRA very suitable for use in on-site or primary medical institutions with limited resources, and only a simple water bath or thermostatic device can be used to complete the detection. However, MIRA also has limitations: it is easy to produce false positive signals, which need to be suppressed by primer design optimization (such as the introduction of base mismatch) (38). Or by optimizing the amount of primers to reduce false positives (39). Susceptibility to inhibitors (e.g., heme, humic acid) in the sample may lead to false negatives (36). The multiple detection ability is still not as good as the mature PCR technology (40, 41).

In this study, high-sensitivity primers were screened from multiple candidate primers for BAstV and BNoV, respectively. After validation of specificity and reproducibility, a duplex MIRA rapid detection method for these two viruses was successfully established. This method was used to detect nucleic acids and plasmids of various bovine diarrhea viruses, including Bovine Rotavirus (BRV), Bovine Viral Diarrhea Virus (BVDV), and Bovine Coronavirus (BCoV). The results showed that only BAstV and BNoV plasmids tested positive, indicating strong specificity and no cross-reactivity with other viruses.

The study found that the sensitivity of the single-plex MIRA assay reached 1 fg/μL for both viruses; however, in the duplex MIRA reaction, the sensitivity decreased to 10 fg/μL and 100 fg/μL, respectively (Figures 7, 8). This result is consistent with previous reports indicating that multiplex MIRA detection faces challenges, and amplification efficiencies among different targets are prone to inconsistency (40, 41). Although the duplex MIRA method established in this study demonstrated satisfactory overall performance, certain limitations must be acknowledged. In this study, the optimization of primer and probe concentrations was primarily conducted using comparable concentrations of BoAstV and BNoV templates. Nevertheless, competitive inhibition-i.e., the suppression of amplification efficiency of a low-concentration target by a high-concentration target—remains an inherent challenge in multiplex nucleic acid detection methods. In this study, we did not extendedly evaluate the potential competitive inhibition effect under mixed sample conditions with varying concentration gradients. Future studies will systematically address this issue to comprehensively assess and further optimize the reaction system, thereby eliminating potential competitive interference.

Clinical sample validation results showed that, compared with the qPCR method, which requires one hour, the developed method could obtain test results in approximately 20 min, and the MIRA assay exhibited a higher detection rate than qPCR. A more in-depth discussion is warranted: this phenomenon cannot be simply attributed to higher analytical sensitivity, as the available data indicate that the analytical sensitivity of the duplex MIRA assay is actually lower than that of the single-plex system. Alternative explanations may involve other factors, including non-specific amplification, false positives, differences in threshold setting, and matrix effects. According to the literature, the RT-MIRA-Cas13a method established by Chuiyu Zhu et al. detected one additional case of hepatitis B virus compared with RT-qPCR, suggesting that false positives might originate from sample transfer contamination or detection system interference (42). Similarly, the CRISPR/Cas13a-based MIRA assay developed by Jiao Fans et al. proposed that false positives might be associated with the setting of the fluorescence threshold (43) Furthermore, isothermal amplification techniques are inherently prone to non-specific amplification, particularly in duplex detection systems. Additionally, the matrix effects of clinical samples may exert stronger inhibition on qPCR, thereby contributing to the observed differences in detection rates between the methods. Future studies will expand the clinical sample size and validate discrepant samples using gold-standard methods (e.g., sequencing) to distinguish true positives from false positives and to further elucidate the underlying mechanisms.

This study not only verified the high efficiency of MIRA technology in detecting BoAstv and BNoV, but also laid a foundation for the expansion of this technology to the detection of RNA pathogens in other animals. The whole process of detection can be completed within 35 min. The equipment is portable and does not depend on the professional laboratory environment. It is suitable for rapid on-site detection. Although MIRA still needs to be improved in multiple detection, this study successfully established a dual detection system with good sensitivity and specificity through primer screening and condition optimization, which provides a fast and sensitive solution for the field of veterinary diagnosis and is expected to promote the overall improvement of pathogen detection level.

## Data Availability

The original contributions presented in the study are included in the article/Supplementary material, further inquiries can be directed to the corresponding author.
