# Supplementary material for: Establishment of duplex multi-enzyme isothermal rapid amplification detection method for bovine astrovirus and norovirus
Source: Front Vet Sci. 2026 Jun 9;13:1819282. doi: 10.3389/fvets.2026.1819282 (PMC13286936; doi:10.3389/fvets.2026.1819282)
Supplement: Supplementary file 1 [file Table_1.doc]

Supplementary Material

# **Supplementary Figures**


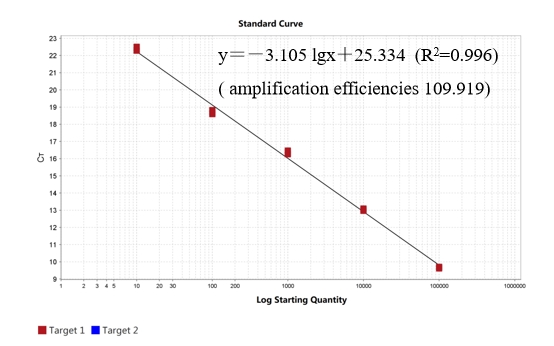


**Supplementary Figure 1**. BoAstV TBgreen standard equation for dye method

The dilution of BoAstV plasmid was 10^-2^,10^-3^,10^-4^,10^-5^,10^-6^, respectively.


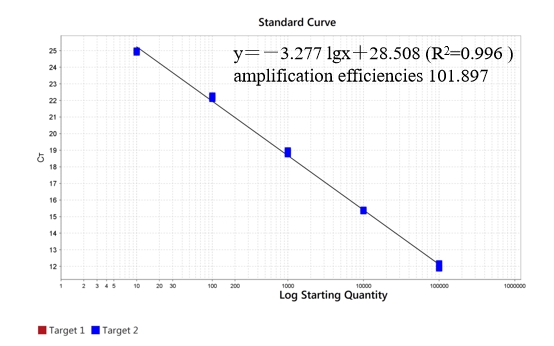


**Supplementary Figure 2**. BNOV TBgreen standard equation for dye method. The dilution of BNOV plasmid was 10^-2^,10^-3^,10^-4^,10^-5^,10^-6^, respectively.


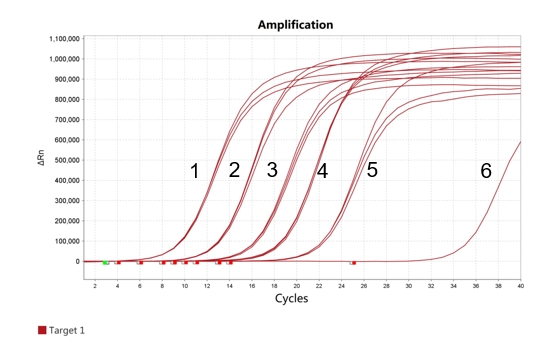


**Supplementary Figure 3**. BoAstV TBgreen dye method amplification curve.

1-5 was BoAstV plasmid 5.1×10^6^copies/µL, 5.1×10^5^copies/µL, 5.1×10^4^copies/µL, 5.1×10^3^copies/µL, 5.1×10^2^copies/µL . 6 was negative control (ddH_2_O) .The detection sensitivity is 5.1×10^2^copies/µL.


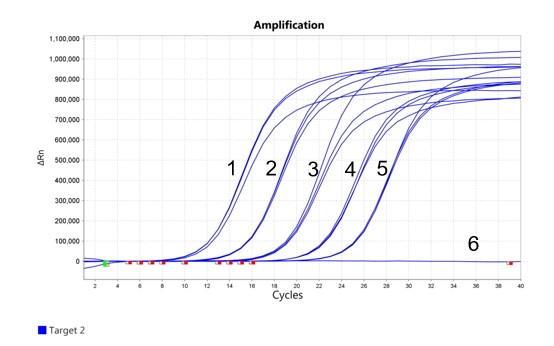


**Supplementary Figure4**. BNOV TBgreen dye method amplification curve. 1-5 was 1.5×10^11^copies/µL,1.5×10^10^copies/µL,1.5×10^9^copies/µL,1.5×10^8^copies/µL,1.5×10^7^copies/µL. 6 was negative control (ddH_2_O) .The detection sensitivity is 1.5×10^7^copies/µL.


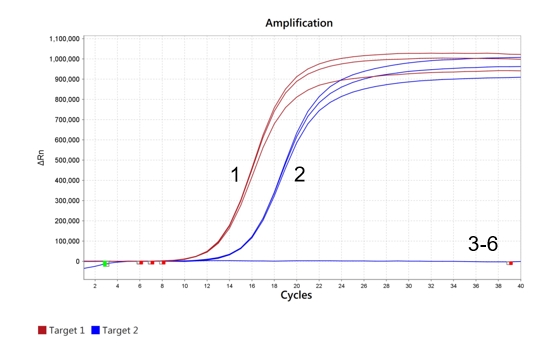


**Supplementary Figure 5.** Specificity of BoAstV and BNOV. 1 BoAstV plasmid, 2 BNOVplasmid. 3-6 plasmid Bovine Rotavirus (BRV), Bovine Viral Diarrhea Virus (BVDV), Bovine Coronavirus (BCoV) and negative control (ddH_2_O).


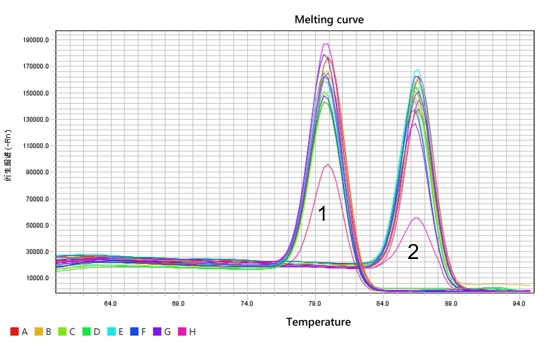


**Supplementary Figure 6**. Melting curve of BoAstV and BNOV. 1 BNOV (79.5℃).

2 BoAstV (86.5℃).

**2.Supplementary Table1.** Reproducibility detection of BoAstV and BNOV TBGreen dual fluorescence

| Virus | Plasmid standard | Intra-assay variability | | Inter-assay variability | |
| --- | --- | --- | --- | --- | --- |
|  | copies/µL | Ct (x̄±S) | *CV*% | Ct (x̄±S) | *CV*% |
| BoAstV | 5.1×10^5^ | 13.03±0.07 | 0.51 | 12.87±0.23 | 1.78 |
|  | 5.1×10^4^ | 16.35±0.11 | 0.67 | 15.59±1.06 | 6.81 |
|  | 5.1×10^3^ | 18.69±0.12 | 0.64 | 18.86±0.23 | 1.21 |
| BNOV | 1.5×10^9^L | 17.79±0.15 | 0.84 | 18.41±0.88 | 4.79 |
|  | 1.5×10^8^ | 21.12±0.08 | 0.39 | 21.67±0.77 | 3.58 |
|  | 1.5×10^7^ | 23.83±0.06 | 0.26 | 24.34±0.72 | 2.96 |
