# Supplementary figures and images for: Establishment of duplex multi-enzyme isothermal rapid amplification detection method for bovine astrovirus and norovirus
Source: Front Vet Sci. 2026 Jun 9;13:1819282. doi: 10.3389/fvets.2026.1819282 (PMC13286936; doi:10.3389/fvets.2026.1819282)

# Amplification Plot

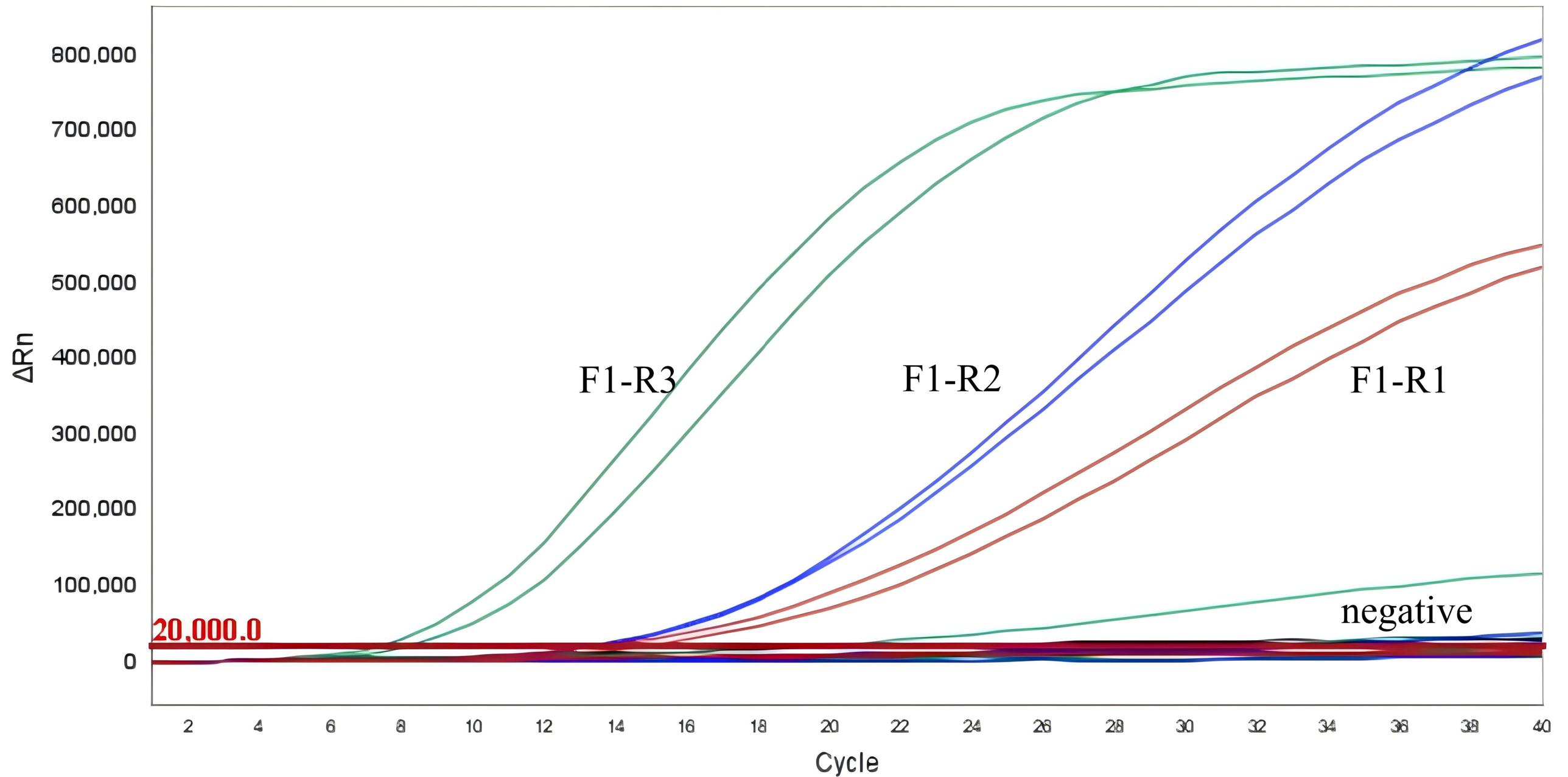

Supplement: Supplementary file 2 [file Supplementary_file_1.zip › fig 1-9/FIG 1.pdf]

**Amplification Plot**

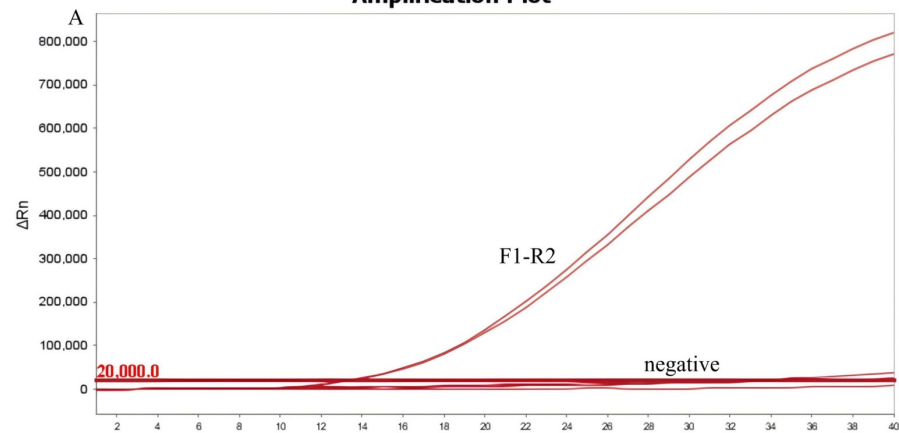

**Amplification Plot**

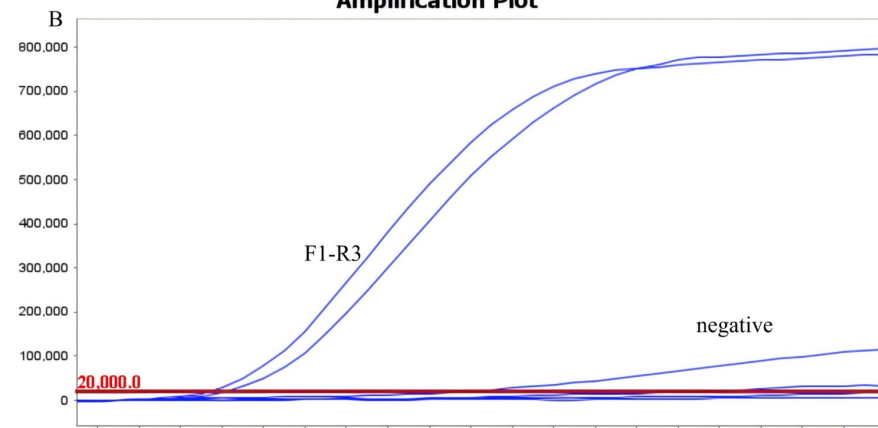

**Amplification Plot**

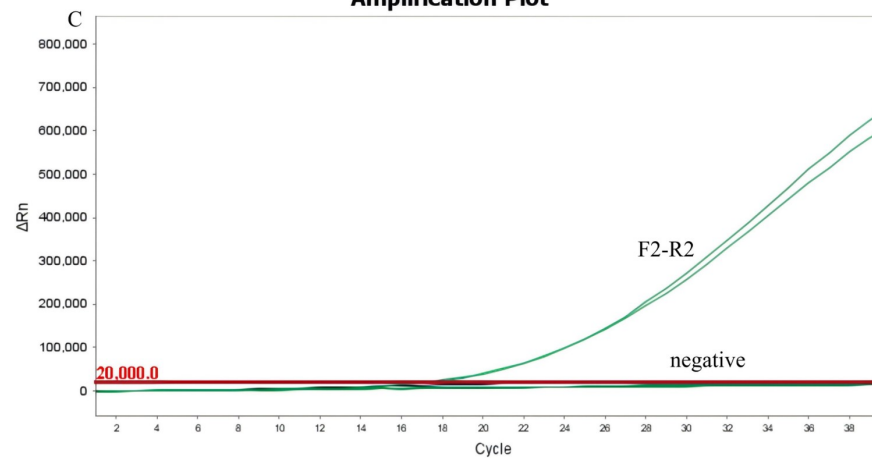

**Amplification Plot**

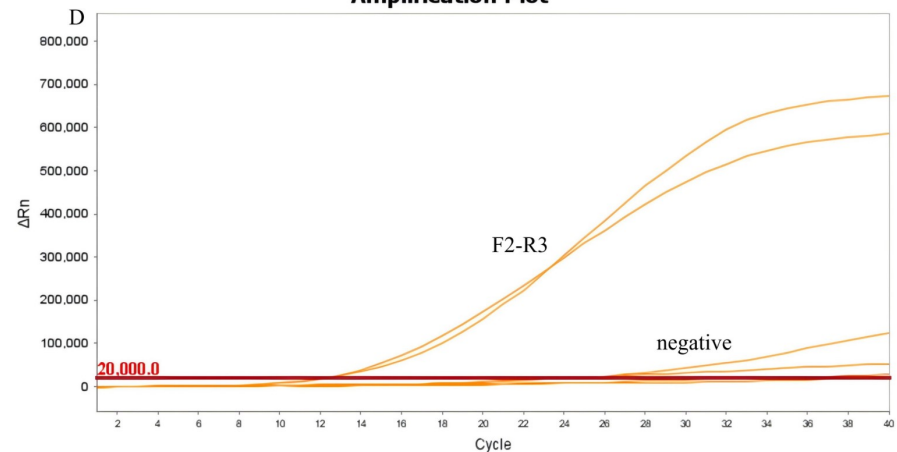

Supplement: Supplementary file 2 [file Supplementary_file_1.zip › fig 1-9/FIG 2.pdf]

# Amplification Plot

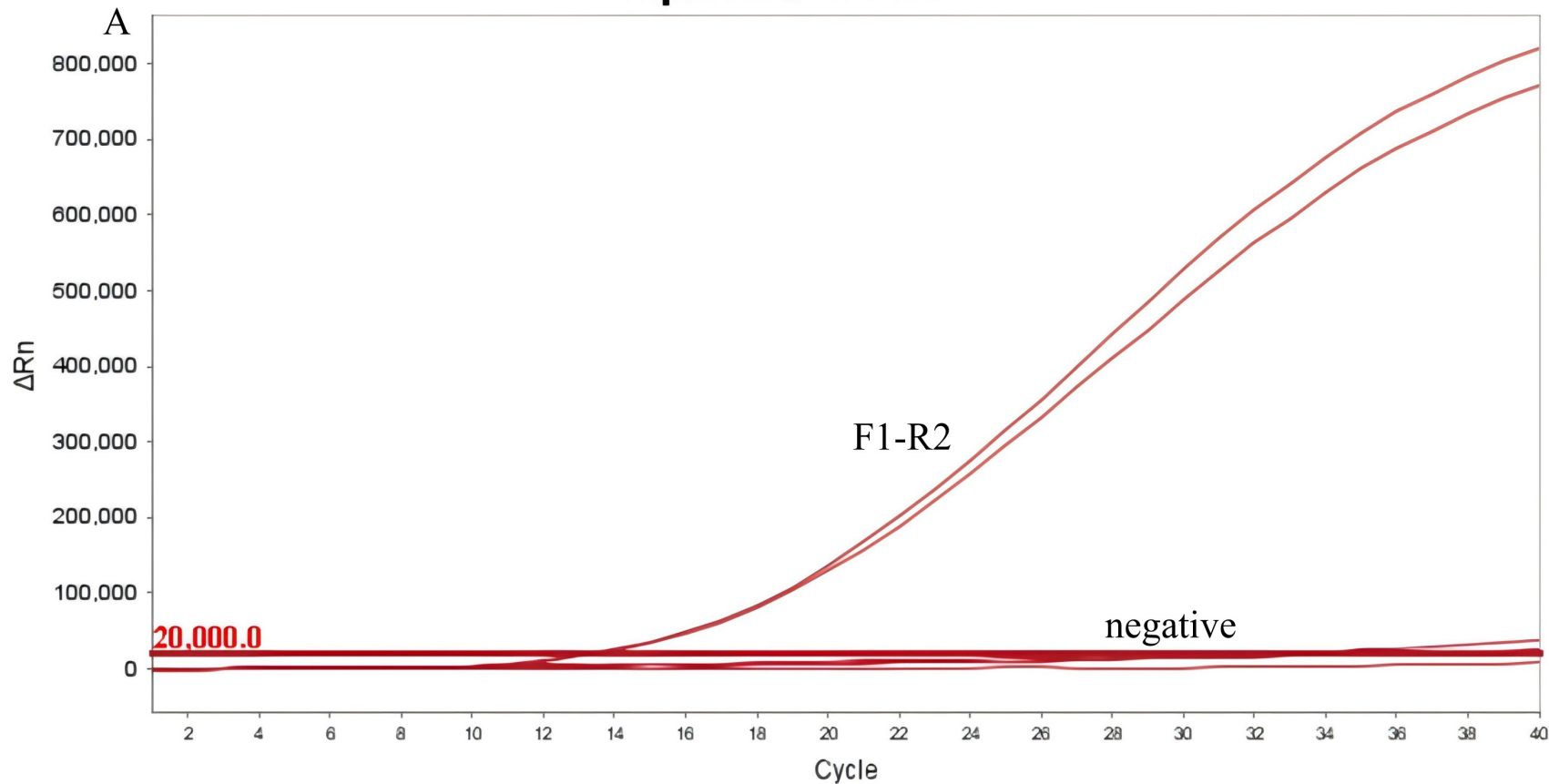

Supplement: Supplementary file 2 [file Supplementary_file_1.zip › fig 1-9/FIG 2A.pdf]

# Amplification Plot

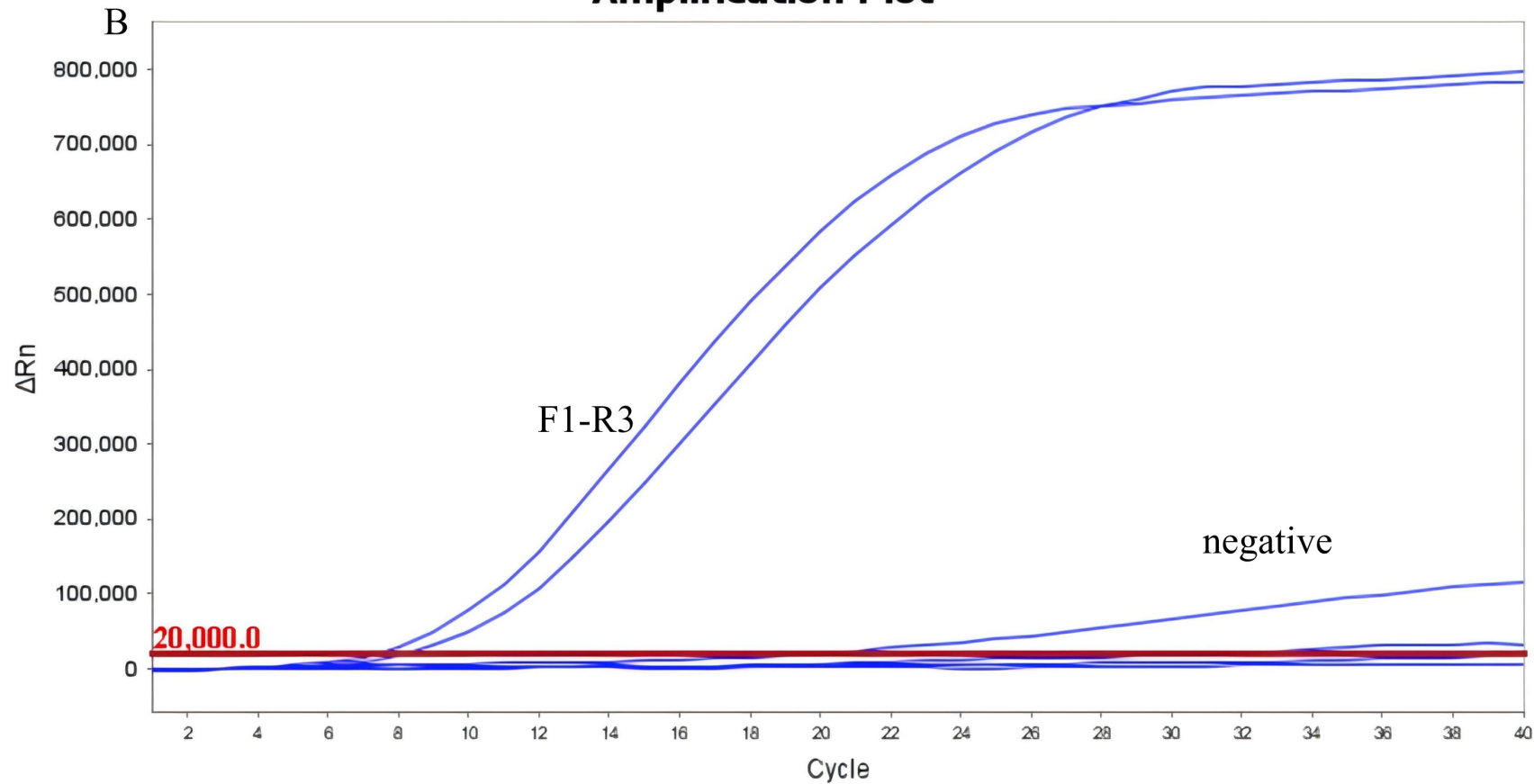

Supplement: Supplementary file 2 [file Supplementary_file_1.zip › fig 1-9/FIG 2B.pdf]

# Amplification Plot

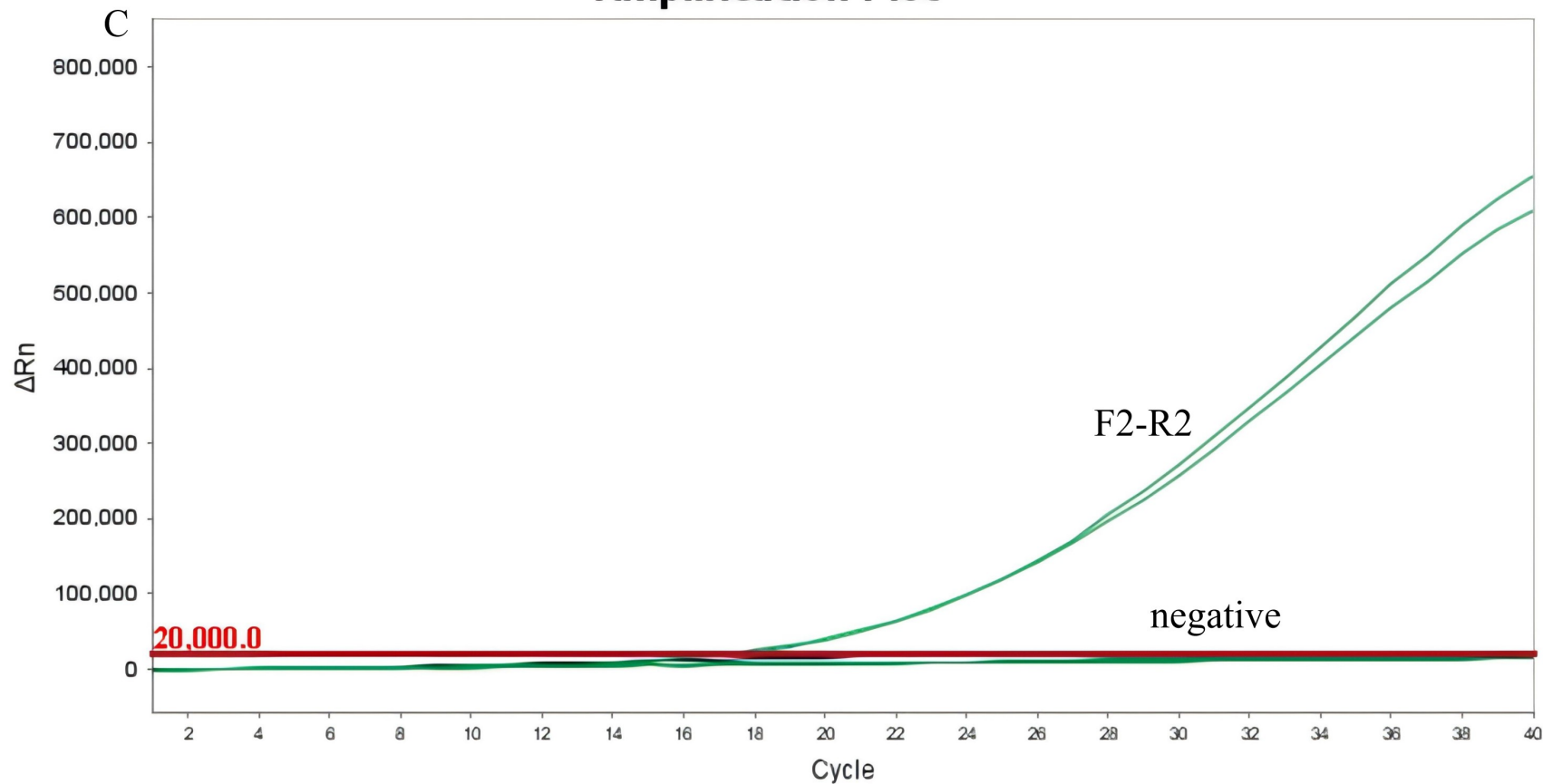

Supplement: Supplementary file 2 [file Supplementary_file_1.zip › fig 1-9/FIG 2C.pdf]

# Amplification Plot

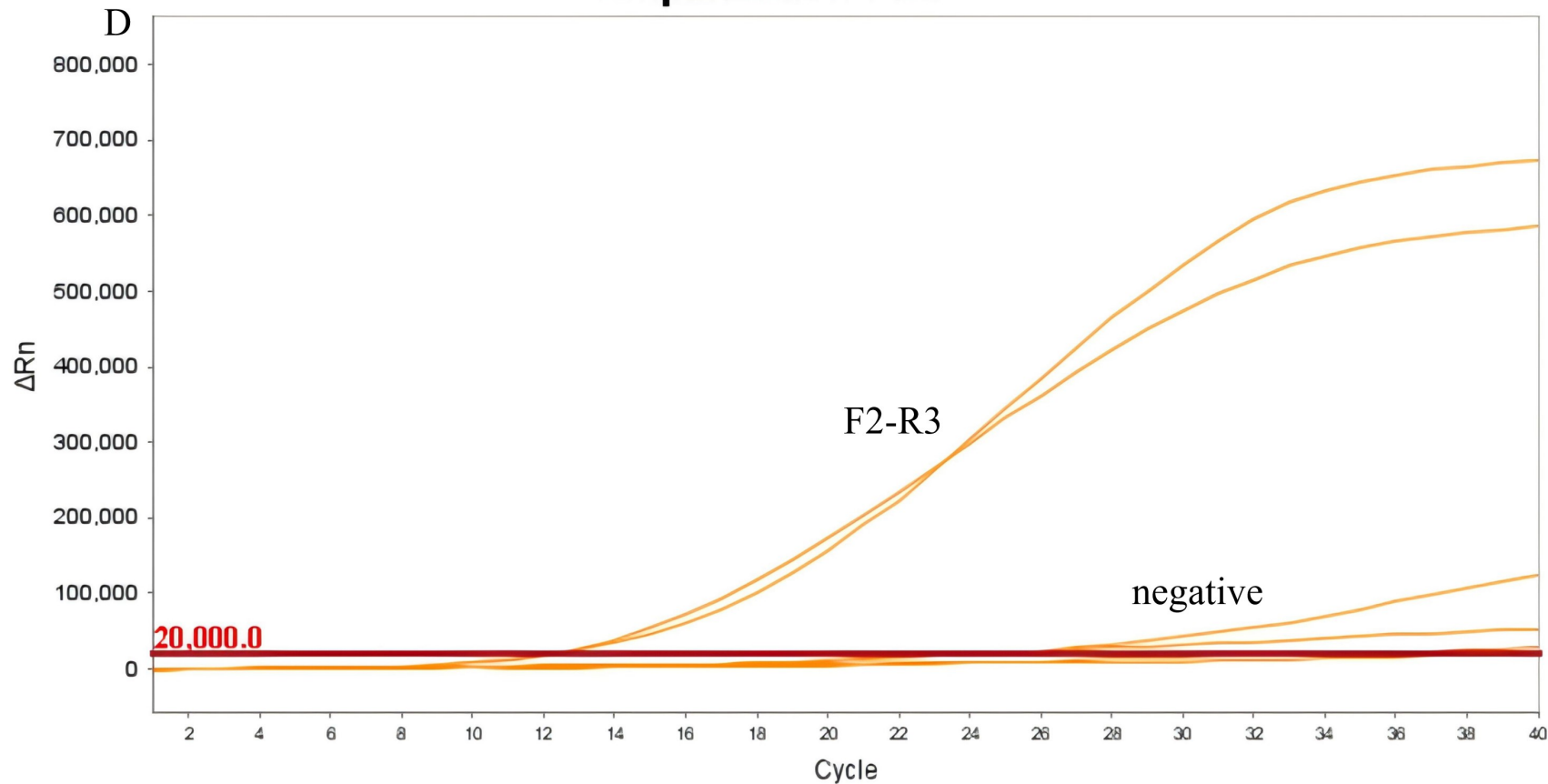

Supplement: Supplementary file 2 [file Supplementary_file_1.zip › fig 1-9/FIG 2D.pdf]

# Amplification Plot

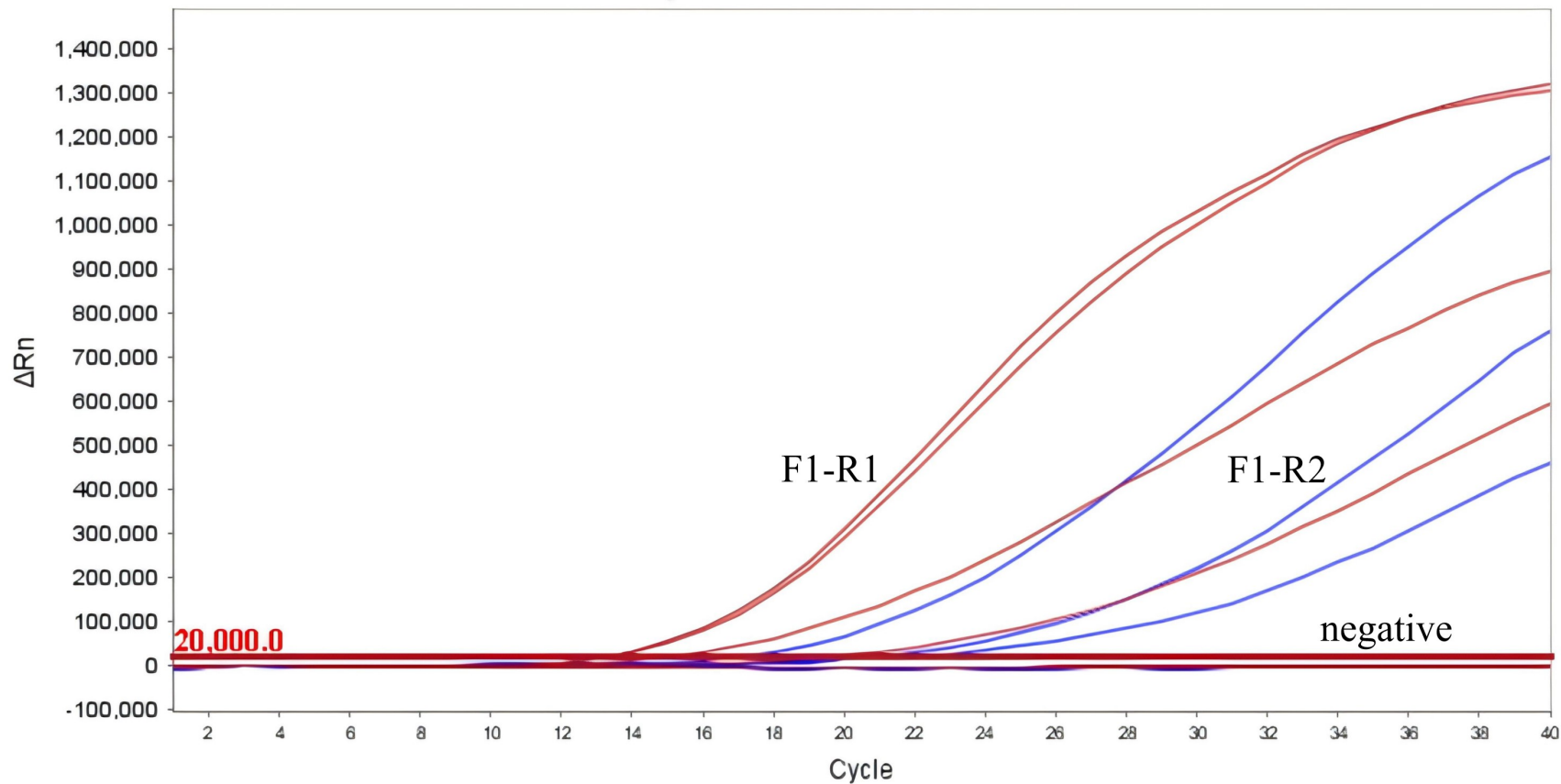

Supplement: Supplementary file 2 [file Supplementary_file_1.zip › fig 1-9/FIG 3.pdf]

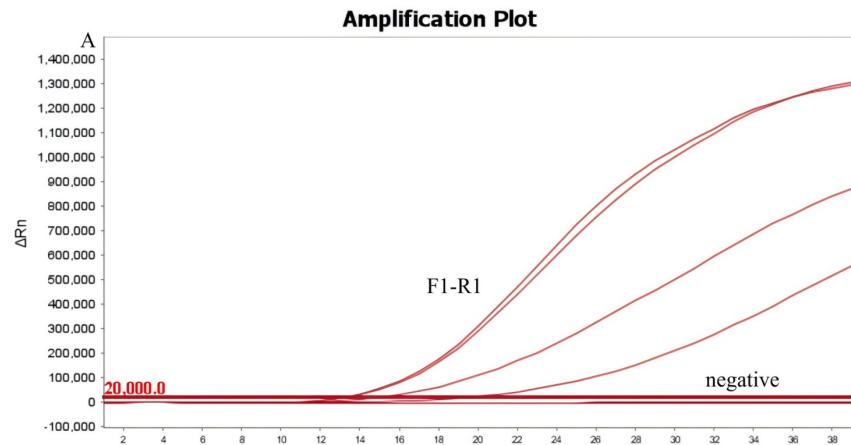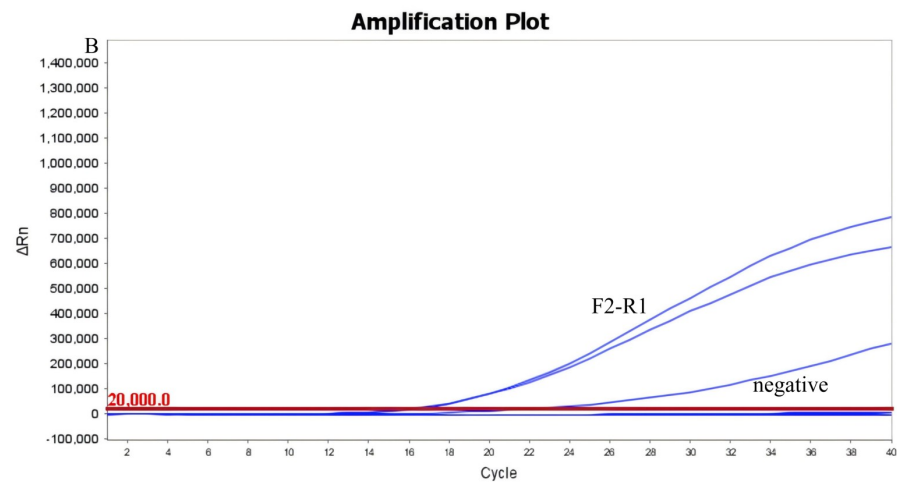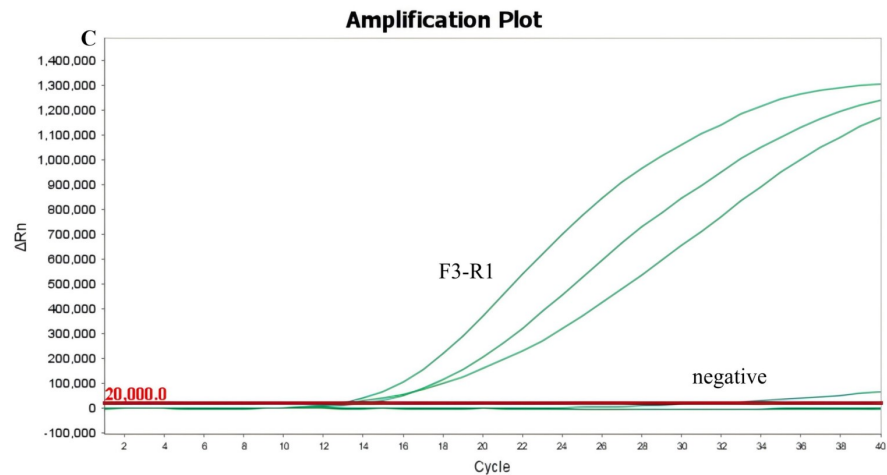

Supplement: Supplementary file 2 [file Supplementary_file_1.zip › fig 1-9/FIG 4.pdf]

# Amplification Plot

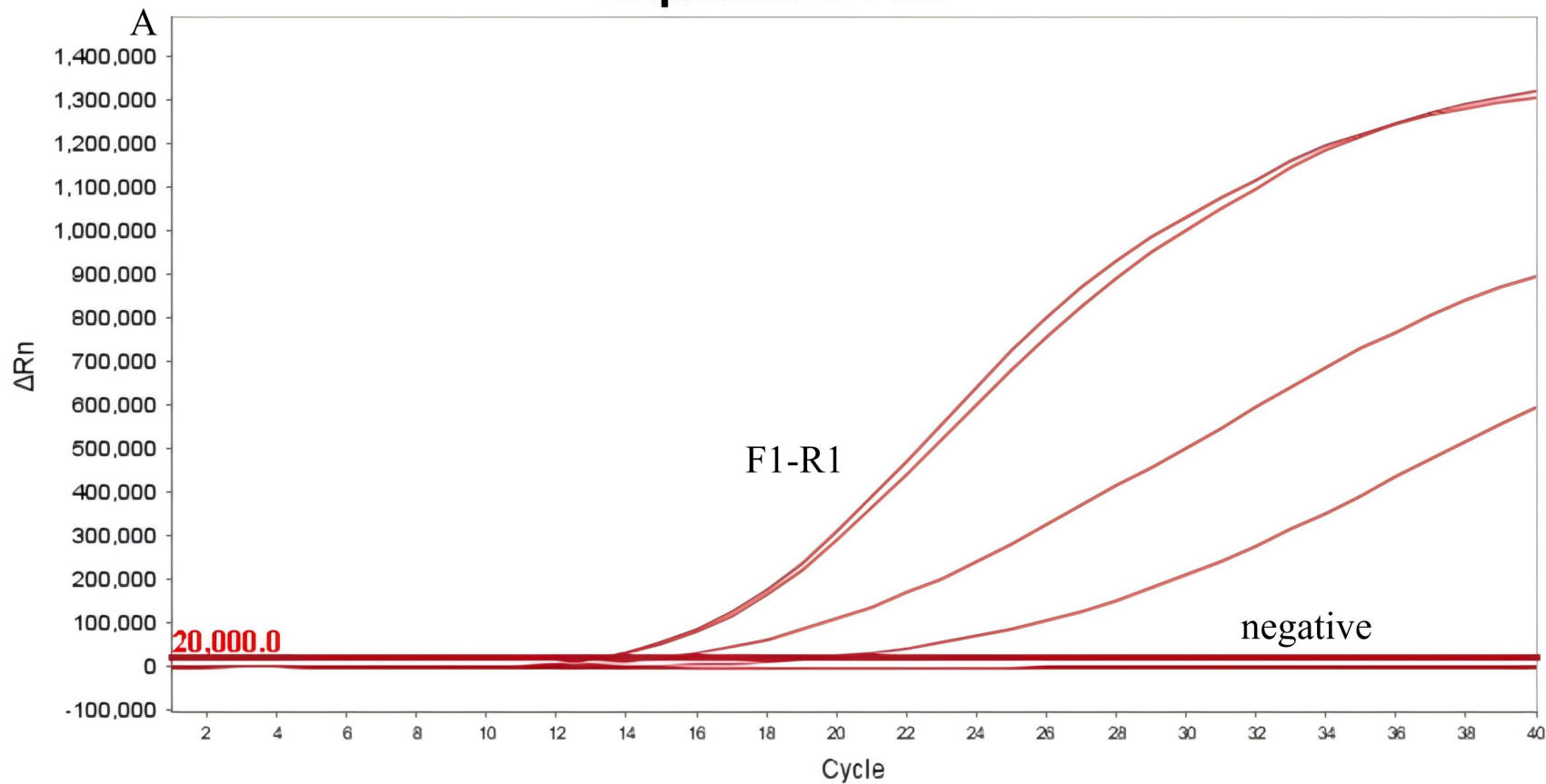

Supplement: Supplementary file 2 [file Supplementary_file_1.zip › fig 1-9/FIG 4A.pdf]

# Amplification Plot

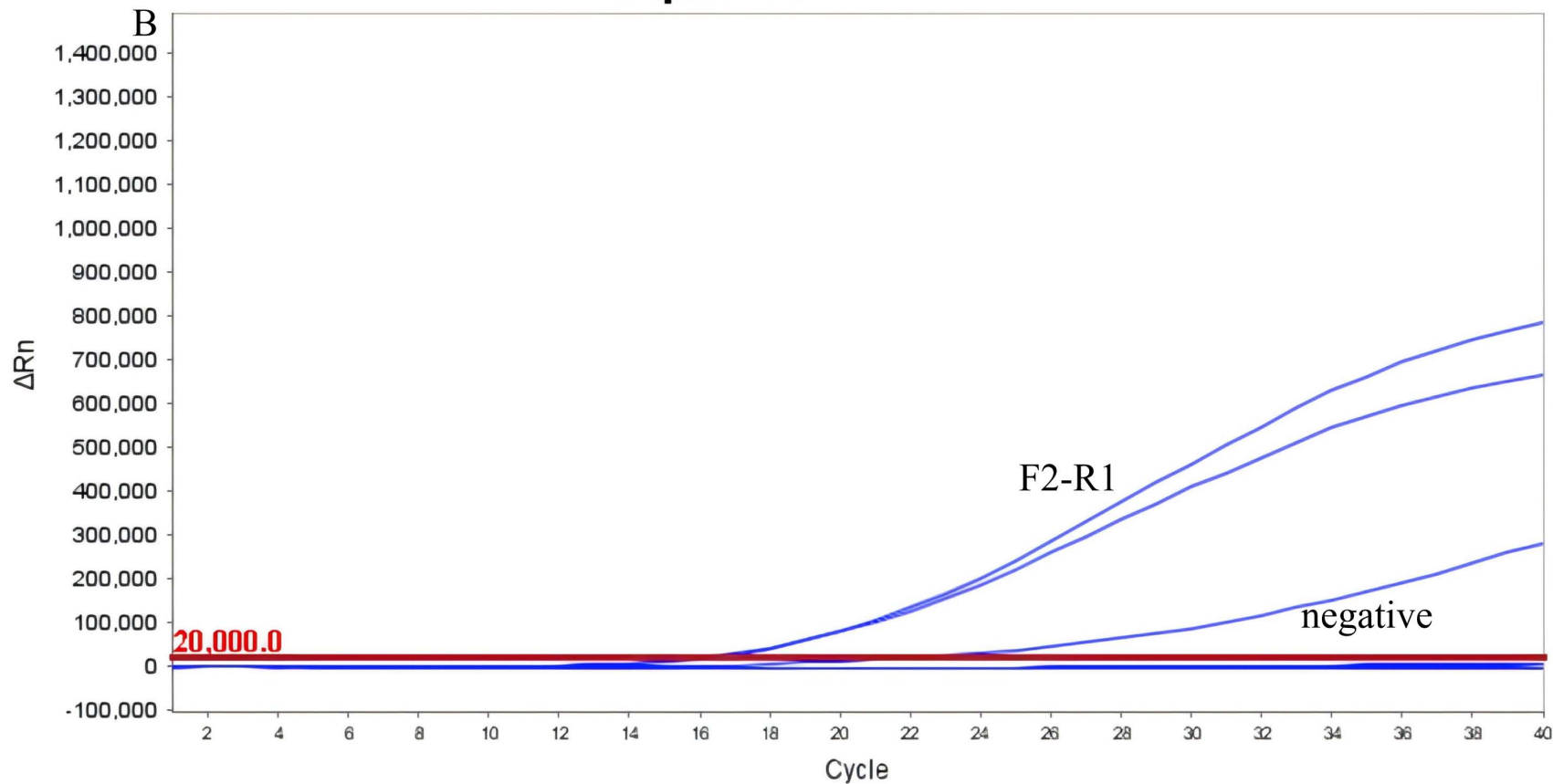

Supplement: Supplementary file 2 [file Supplementary_file_1.zip › fig 1-9/FIG 4B.pdf]

# Amplification Plot

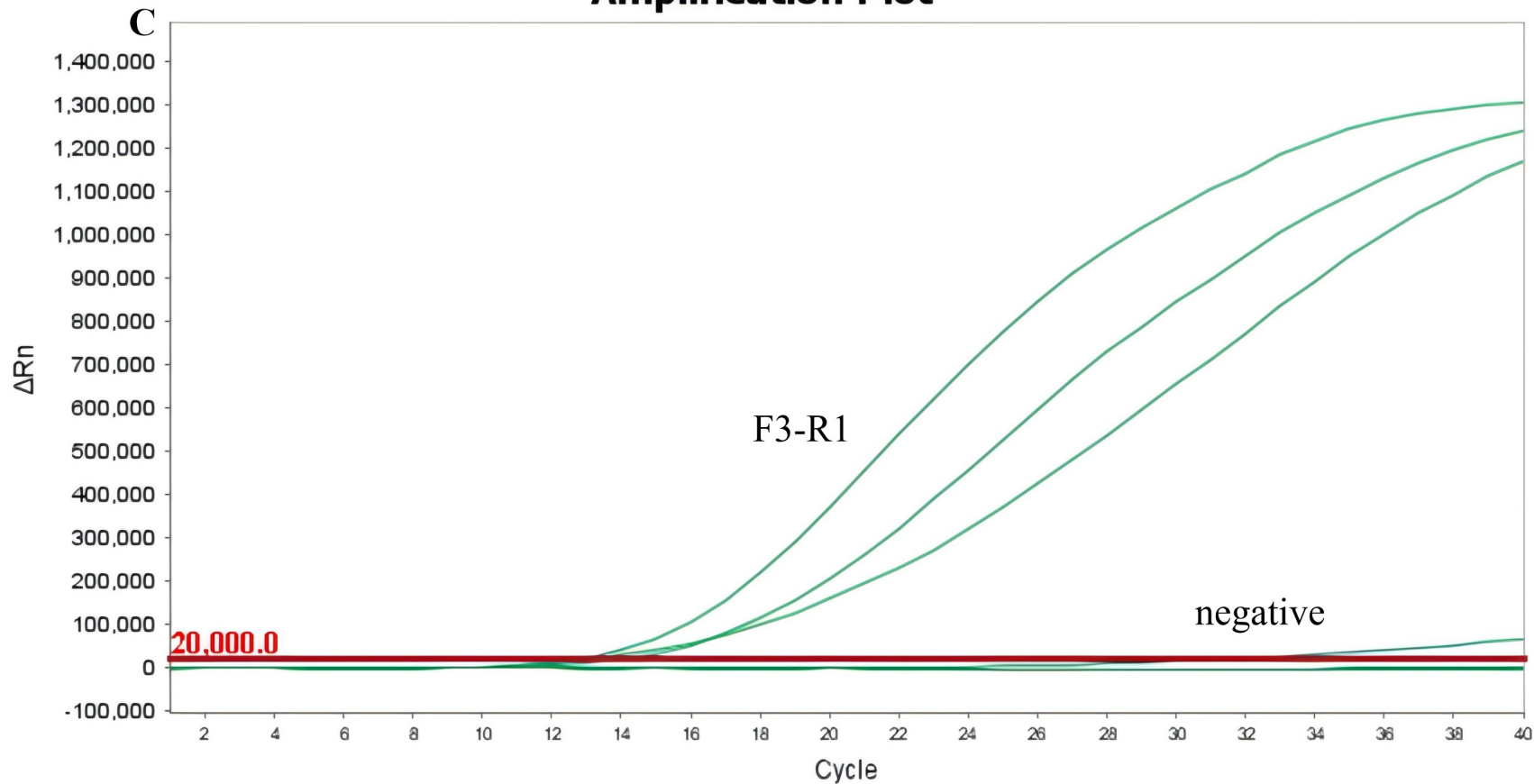

Supplement: Supplementary file 2 [file Supplementary_file_1.zip › fig 1-9/FIG 4C.pdf]

# Amplification Plot

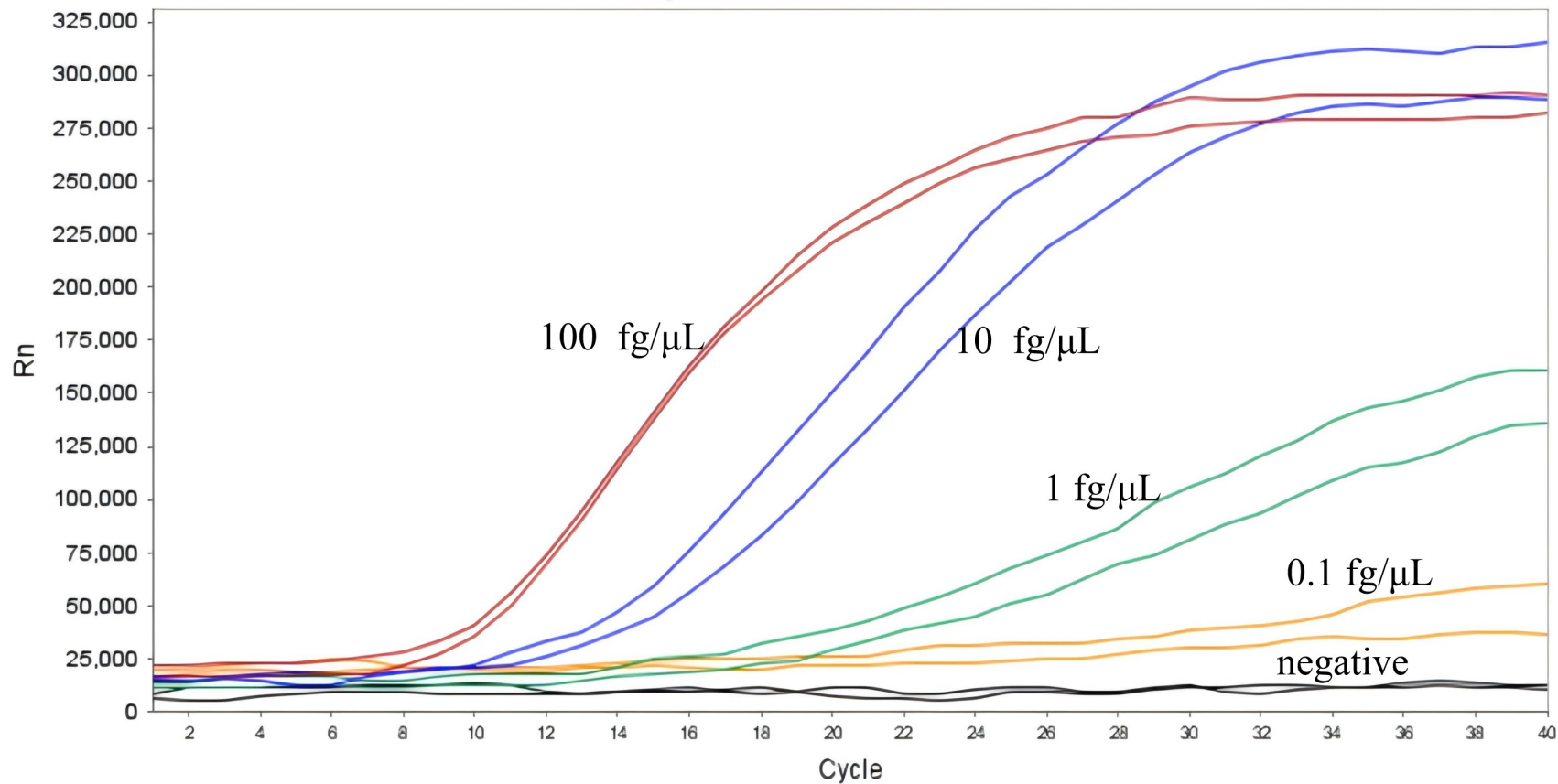

Supplement: Supplementary file 2 [file Supplementary_file_1.zip › fig 1-9/FIG 5.pdf]

### Amplification Plot

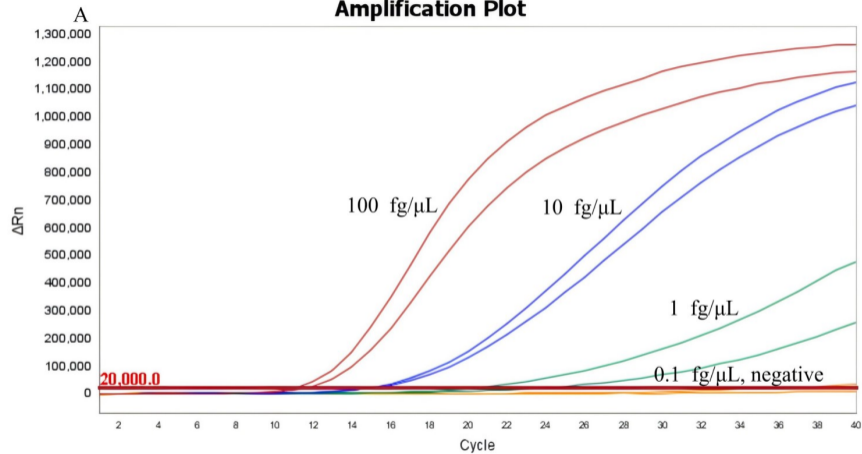

### Amplification Plot

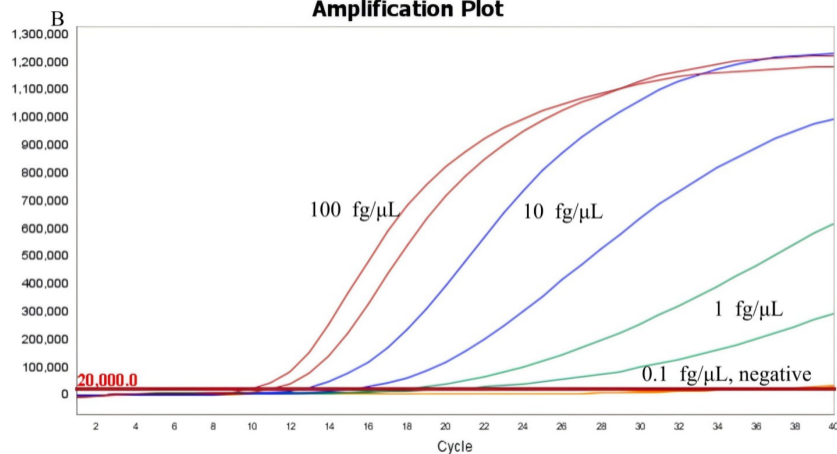

Supplement: Supplementary file 2 [file Supplementary_file_1.zip › fig 1-9/FIG 6.pdf]

# Amplification Plot

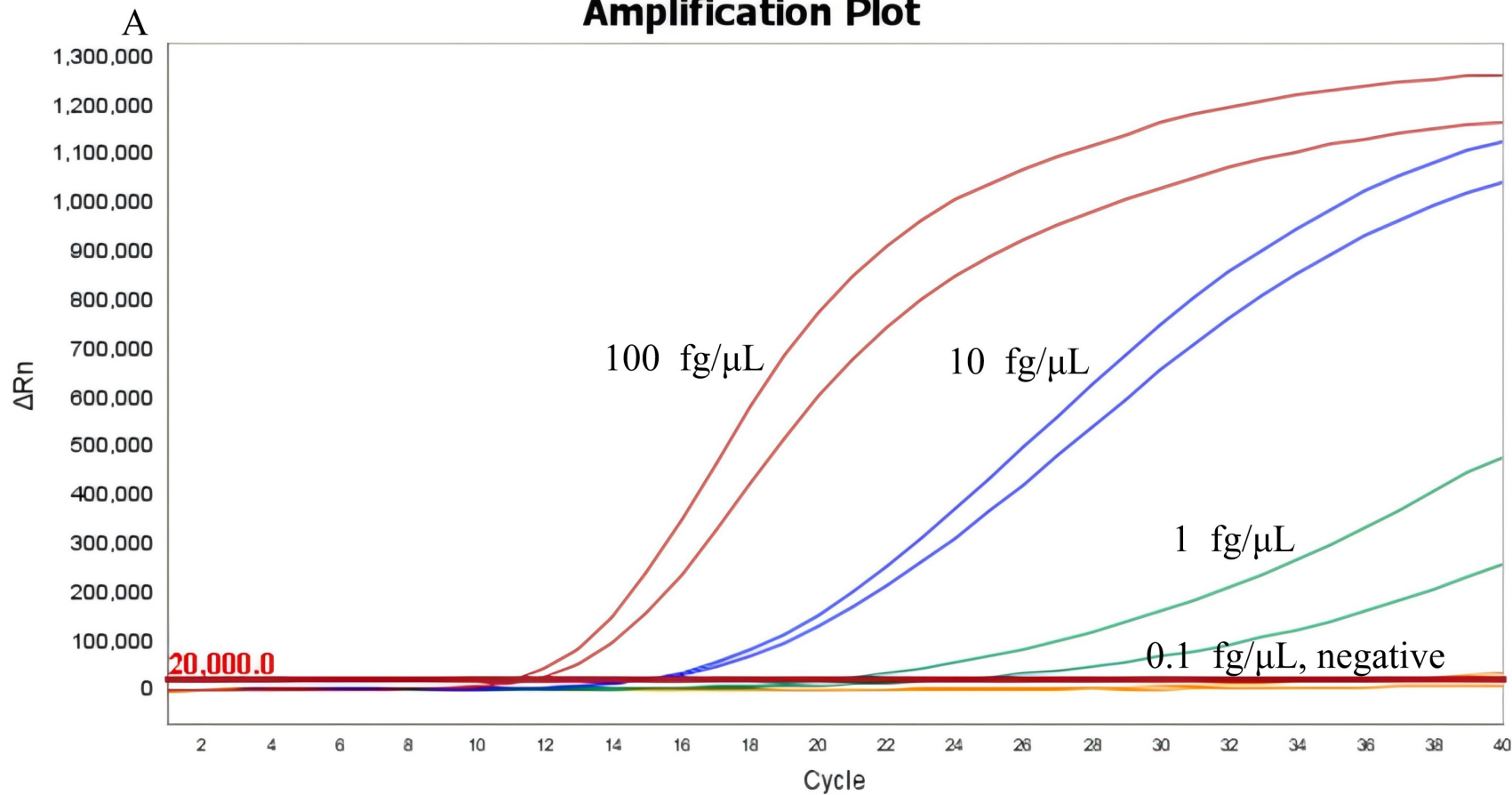

Supplement: Supplementary file 2 [file Supplementary_file_1.zip › fig 1-9/FIG 6A.pdf]

# Amplification Plot

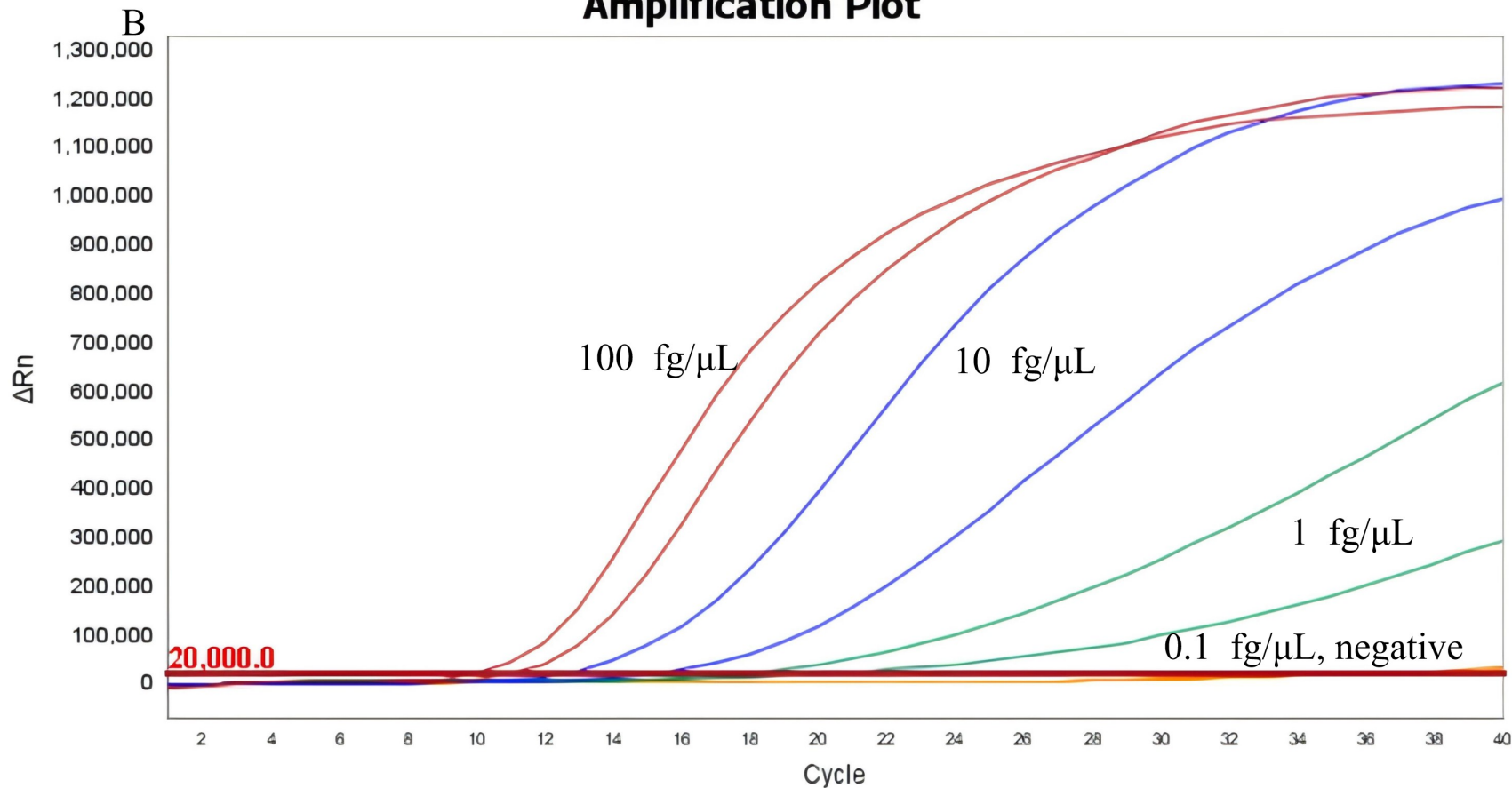

Supplement: Supplementary file 2 [file Supplementary_file_1.zip › fig 1-9/FIG 6B.pdf]

**Amplification Plot**

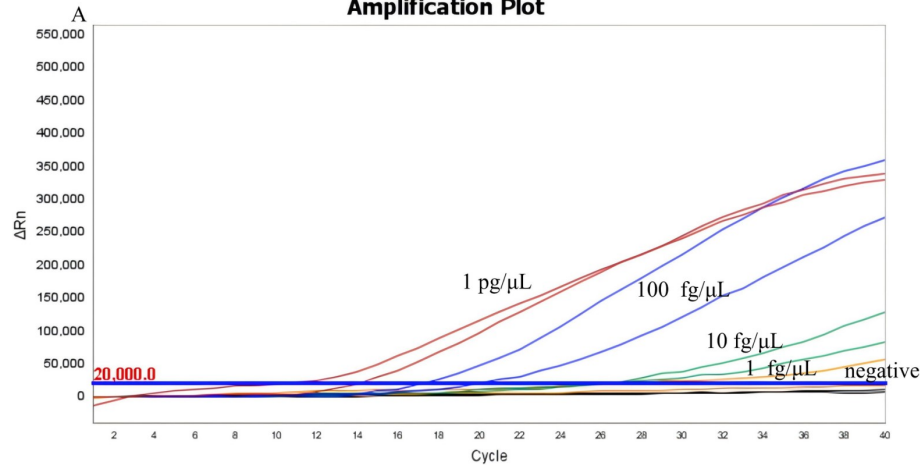

**Amplification Plot**

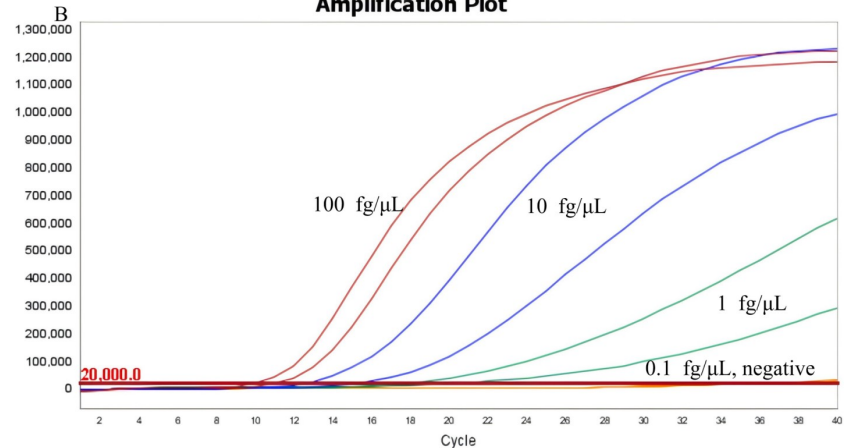

Supplement: Supplementary file 2 [file Supplementary_file_1.zip › fig 1-9/FIG 7.pdf]

# Amplification Plot

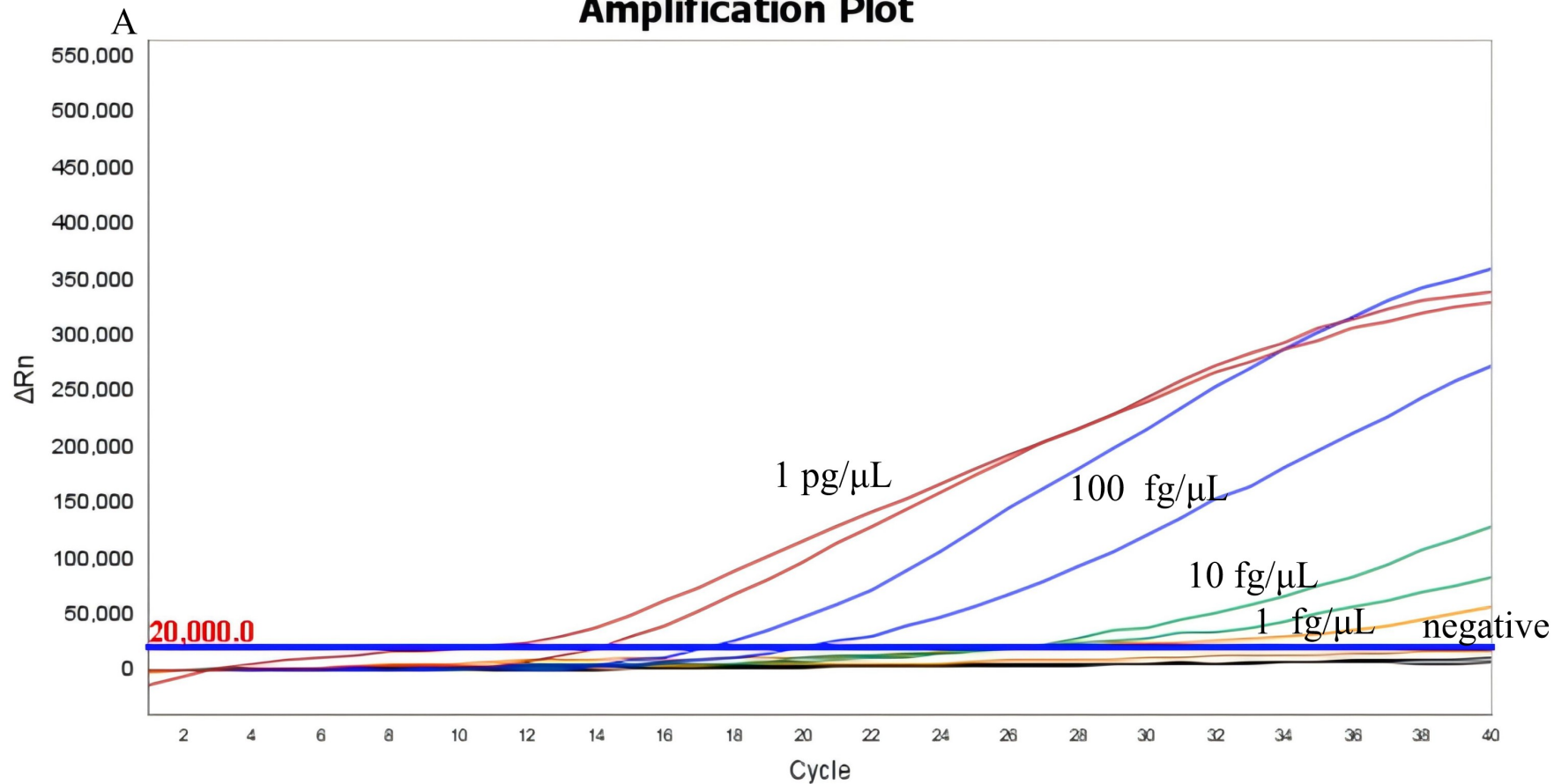

Supplement: Supplementary file 2 [file Supplementary_file_1.zip › fig 1-9/FIG 7A.pdf]

# Amplification Plot

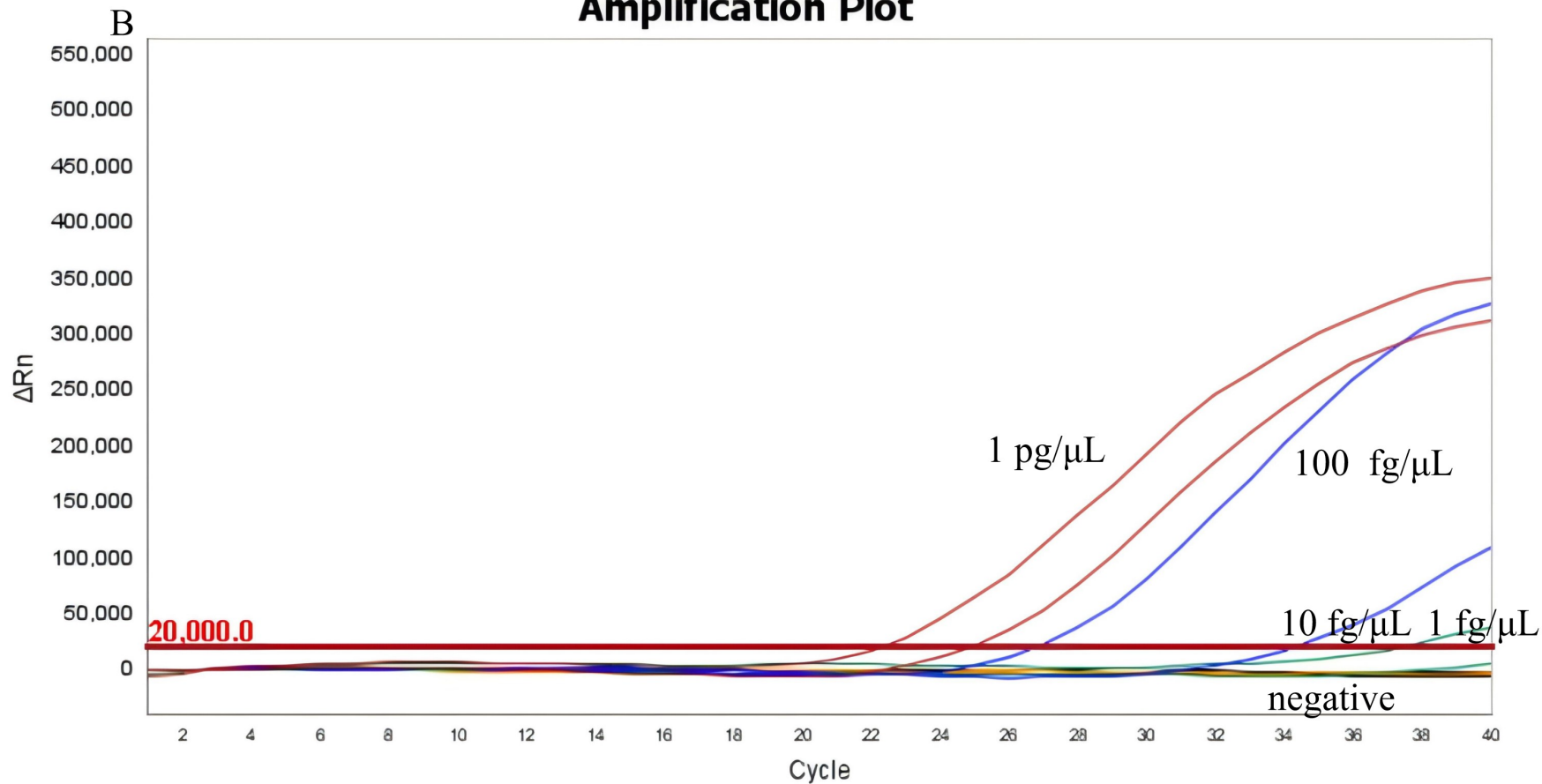

Supplement: Supplementary file 2 [file Supplementary_file_1.zip › fig 1-9/FIG 7B.pdf]

### Amplification Plot

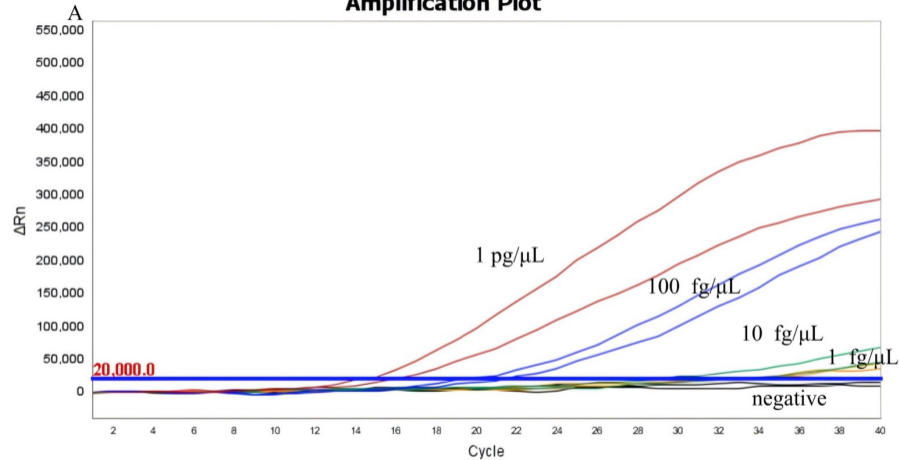

### Amplification Plot

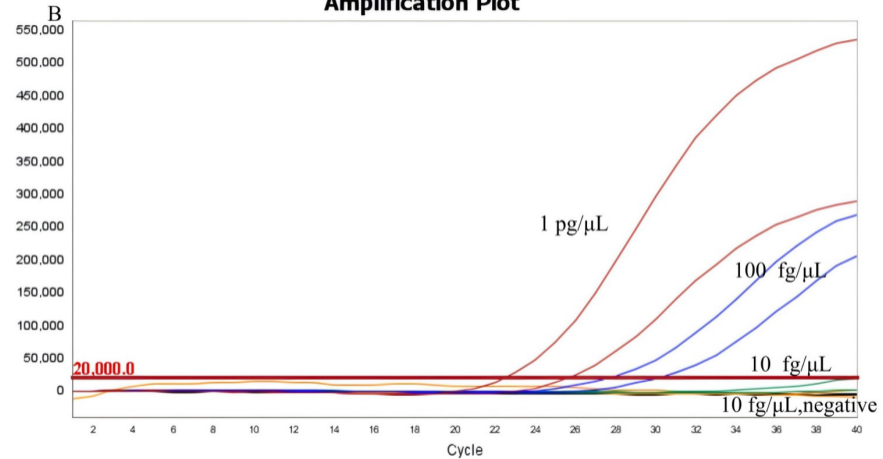

Supplement: Supplementary file 2 [file Supplementary_file_1.zip › fig 1-9/FIG 8.pdf]

# Amplification Plot

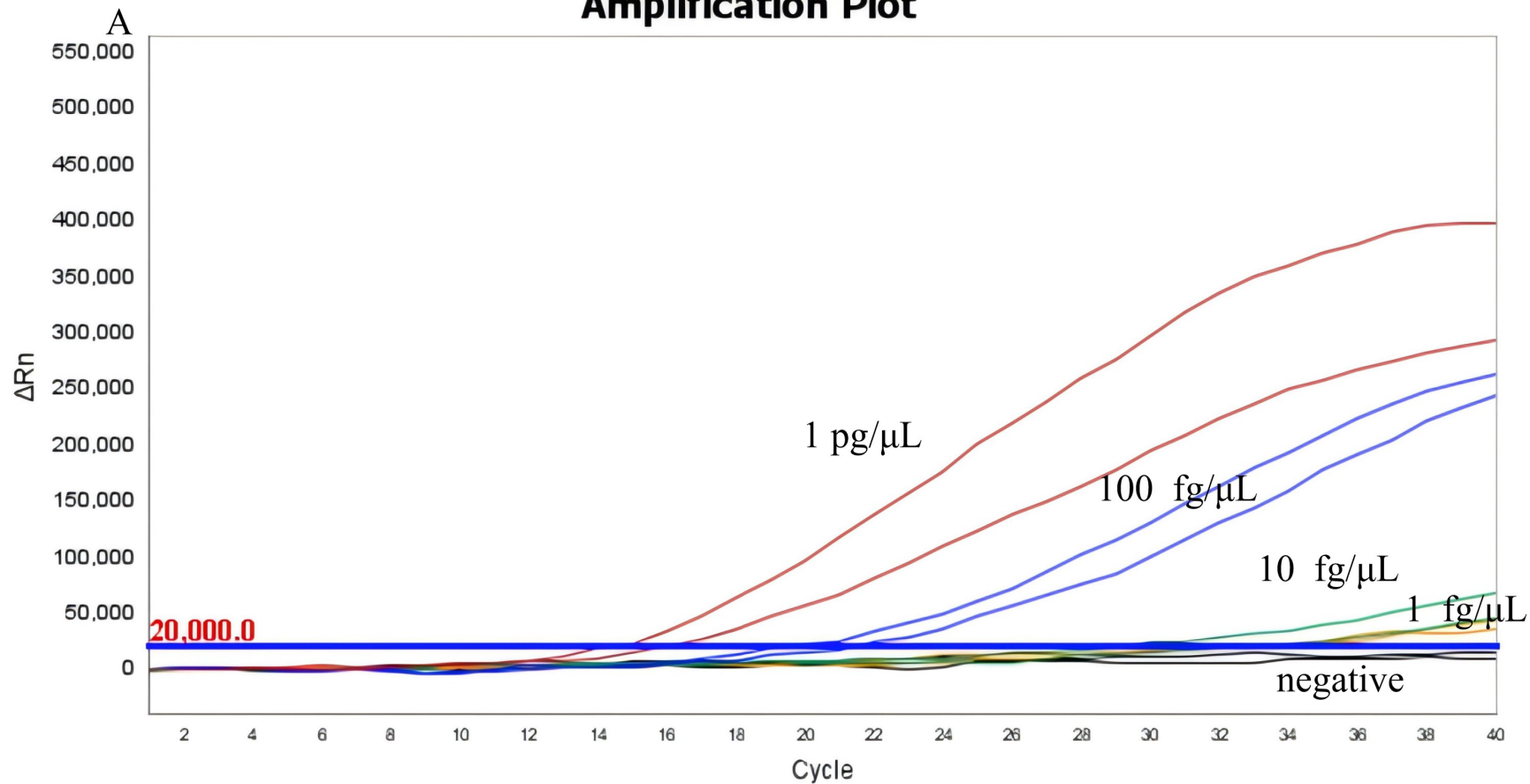

Supplement: Supplementary file 2 [file Supplementary_file_1.zip › fig 1-9/FIG 8A.pdf]

# Amplification Plot

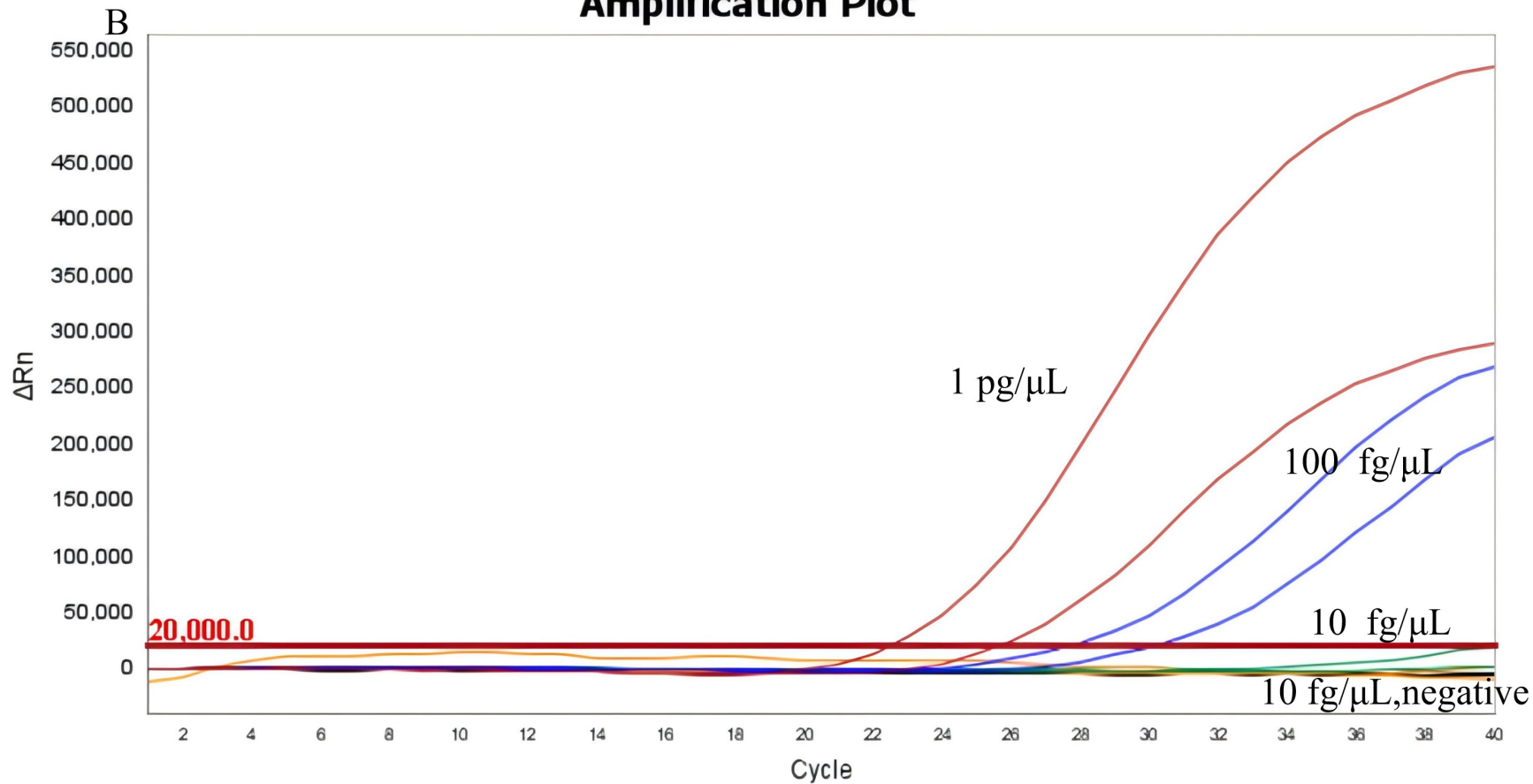

Supplement: Supplementary file 2 [file Supplementary_file_1.zip › fig 1-9/FIG 8B.pdf]
