# Supplementary material for: Establishment of duplex multi-enzyme isothermal rapid amplification detection method for bovine astrovirus and norovirus
Source: Front Vet Sci. 2026 Jun 9;13:1819282. doi: 10.3389/fvets.2026.1819282 (PMC13286936; doi:10.3389/fvets.2026.1819282)
Supplement: Supplementary file 2 [file FIG 9.pdf › fig 1-9/FIG 9.pdf]

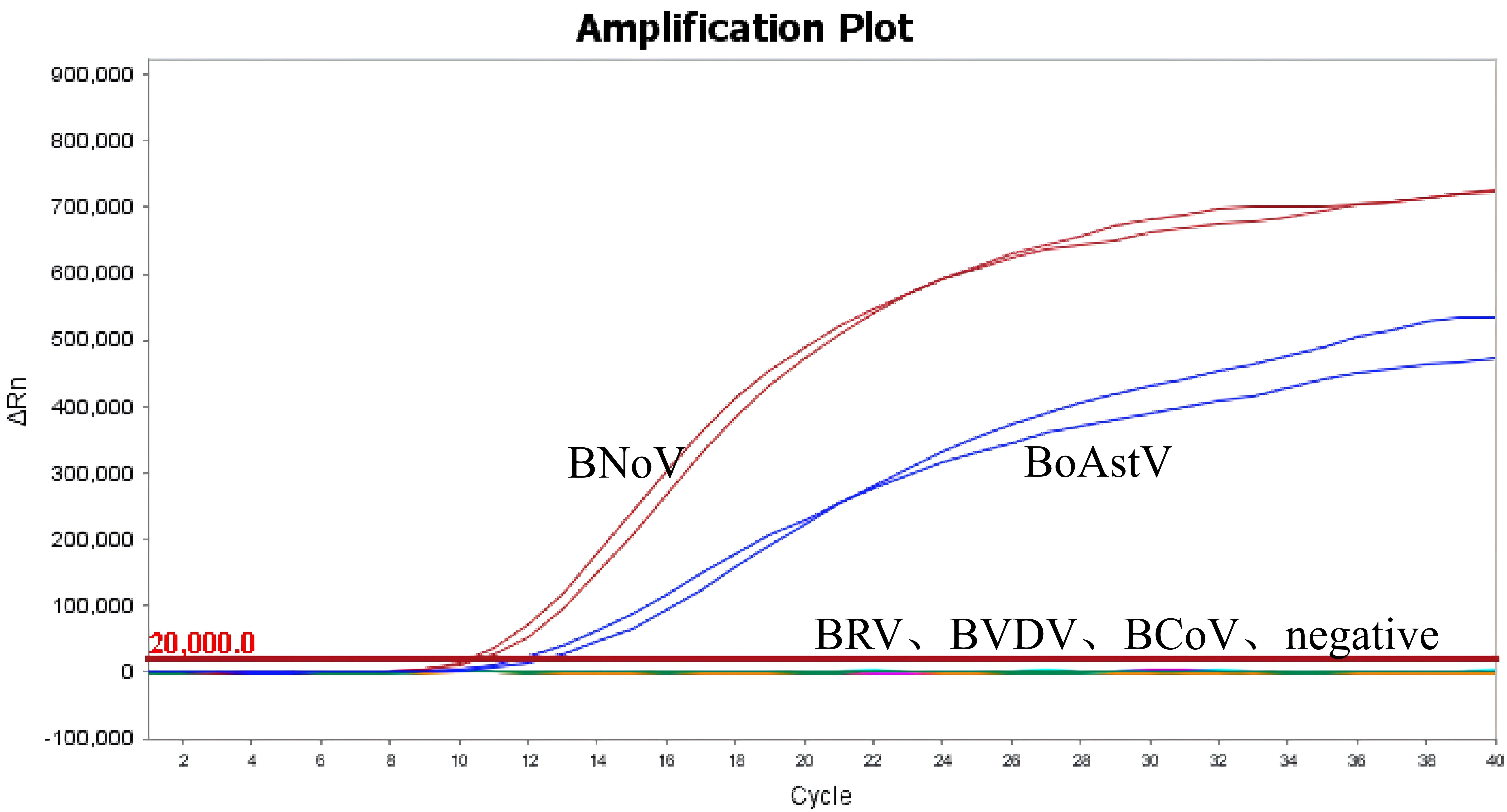
Amplification Plot
900,000
800,000
700,000
600,000
500,000
400,000
300,000
200,000
100,000
0
-100,000
ΔRn
20,000.0
BNoV
BoAstV
BRV、BVDV、BCoV、negative
2
4
6
8
10
12
14
16
18
20
22
24
26
28
30
32
34
36
38
40
Cycle
